# Targeting the PGRN‐BMP Lysosomal Axis With NPs@PGRN Reverses Immunometabolic Dysfunction in Chronic Septic Arthritis

**DOI:** 10.1002/advs.202512133

**Published:** 2026-03-16

**Authors:** Congsun Li, Jiaqi Fan, Tao Sun, Wushi Cui, Jiaxuan Zou, Weinan Yang, Liang Chen, Jian Xiao, Shicheng Wang, Tao Zhang, Jianqiao Hong, An Liu, Haobo Wu

**Affiliations:** ^1^ Department of Orthopedics, The Second Affiliated Hospital Zhejiang University School of Medicine Hangzhou Zhejiang P. R. China; ^2^ Orthopedics Research Institute of Zhejiang University Hangzhou Zhejiang P. R. China; ^3^ Key Laboratory of Motor System Disease Research and Precision Therapy of Zhejiang Province, The Second Affiliated Hospital Zhejiang University Hangzhou Zhejiang P. R. China; ^4^ Clinical Research Center of Motor System Disease of Zhejiang Province Hangzhou Zhejiang P. R. China; ^5^ State Key Laboratory of Transvascular Implantation Devices Hangzhou P. R. China; ^6^ Zhejiang Key Laboratory of Smart Biomaterials, Key Laboratory of Biomass Chemical Engineering of Ministry of Education, College of Chemical and Biological Engineering Zhejiang University Hangzhou P. R. China

**Keywords:** immune function, infection, lipid metabolism, lysosome, macrophage, nanoparticle

## Abstract

Chronic septic arthritis, often progressing to refractory infection due to intracellular bacterial persistence, is accompanied by dynamic macrophage immunometabolic reprogramming. Prolonged intracellular bacterial survival drives lipid metabolic remodeling in macrophages, leading to progressive accumulation of bis(monoacylglycero)phosphate (BMP), regulated by progranulin (PGRN). Mechanistically, PGRN maintains lysosomal homeostasis through direct interaction with BMP. PGRN deficiency disrupts lysosomal integrity, induces immunosuppressive polarization, and facilitates bacterial immune evasion. To address this, a dual‐targeting nanoparticle system (NPs@PGRN) targeting the “PGRN‐BMP‐lysosome” axis was developed. NPs@PGRN restores lysosomal bactericidal capacity via PGRN replenishment, significantly reduces bacterial burdens, and reprograms macrophage immunophenotypes toward antimicrobial competence. This study elucidates the central role of lipid‐immune crosstalk in chronic joint infection pathogenesis and provides a novel therapeutic strategy for it.

## Graphical abstract

Chronic septic arthritis involves intracellular bacterial persistence and lipid‐immune crosstalk via the PGRN‐BMP lysosomal axis. A dual‐targeting nanoparticle system (NPs@PGRN) restores lysosomal bactericidal function, reduces bacterial burden, and reprograms macrophage immunity, offering a novel therapeutic strategy.

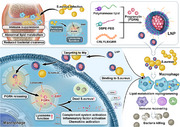

## 1 Introduction

Bacterial infections of bone and joint tissue remain a significant challenge in orthopedic practice. The limited blood supply to osseous structures impedes adequate delivery of antibiotics and effector immune cells to infected sites [1]. Furthermore, pathogens such as Staphylococcus aureus (*S.aureus*) and specific Gram‐negative (G**^−^**) bacteria employ endocytic mechanisms to invade host cells, thereby evading both immune surveillance and antimicrobial activity. These intracellular pathogens demonstrate remarkable capacity for prolonged intracellular survival within host cellular compartments [2], ultimately leading to refractory chronic septic arthritis.

Macrophages (Mφ) are pivotal immune cells that play critical roles during infection. Under physiological conditions, macrophages recognize and phagocytose pathogens such as bacteria via surface receptors, initiating primary immune responses. Concurrently, they secrete inflammatory cytokines to recruit additional immune cells to the infection site, amplifying local immune activation [3]. Following pathogen clearance, macrophages transition to secreting growth factors and extracellular matrix components, mediating tissue repair and regeneration [4]. This dynamic functional transition highlights the temporal evolution of macrophage immunobiology in septic arthritis‐from robust pro‐inflammatory responses during acute infection to immunosuppressive phenotypes in chronic stages‐with profound implications for infection resolution.

Under normal circumstances, following bacterial invasion, damaged tissues release inflammatory mediators that recruit macrophages to the infection site. These macrophages recognize pathogen‐associated molecular patterns (PAMPs), such as lipopolysaccharides and peptidoglycans, through surface receptors, thereby initiating phagocytosis [5, 6, 7]. The macrophages extend pseudopodia via membrane protrusion to engulf bacteria, forming internalized vesicles called phagosomes. Subsequently, these phagosomes fuse with intracellular lysosomes to generate phagolysosomes. Lysosomal enzymes, including lysozyme and proteases, are released to directly degrade bacterial cell walls and intracellular components [8, 9]. Concurrently, macrophages produce reactive oxygen species (ROS) and nitric oxide (NO) to damage bacterial DNA, proteins, and membrane structures [3, 10]. The acidic environment within phagolysosomes (pH reduction) further suppresses bacterial viability [9, 11, 12]. However, during persistent infections, these bactericidal mechanisms of macrophages appear compromised. Specifically, macrophage immune functions become suppressed, rendering them unable to fully eliminate internalized bacteria. Consequently, macrophages may serve as sanctuaries for bacterial evasion of both immune surveillance and antimicrobial therapies.

Under infection or inflammatory conditions, macrophages undergo metabolic reprogramming to adapt to environmental changes, altering their cytokine secretion profiles and thereby modulating the intensity and nature of immune responses [13, 14, 15, 16, 17, 18, 19]. In the context of persistent bone infections, sustained immune activation leads to functional suppression of macrophages, impairing their pathogen clearance capacity and compromising overall immune responses [20, 21], which may contribute to the establishment of an immunosuppressive microenvironment [22, 23]. M1‐polarized macrophages typically rely on glycolysis and fatty acid oxidation to fuel pro‐inflammatory cytokine production, whereas M2‐polarized macrophages predominantly utilize lipid synthesis and oxidation to mediate tissue repair and anti‐inflammatory responses. The lipid metabolic status of macrophages has been shown to influence their polarization patterns [24, 25]; however, the precise molecular mechanisms and key regulatory metabolites involved remain incompletely understood.

Collectively, although multiple mechanisms underlying intracellular bacteria‐mediated modulation of macrophage functions have been reported, existing studies predominantly focus on cross‐sectional timepoints, lacking longitudinal comparative analyses across different infection phases. Therefore, the temporal dynamics of macrophage functional alterations following bacterial phagocytosis and the molecular drivers of these changes warrant systematic investigation.

**SCHEME 1:**
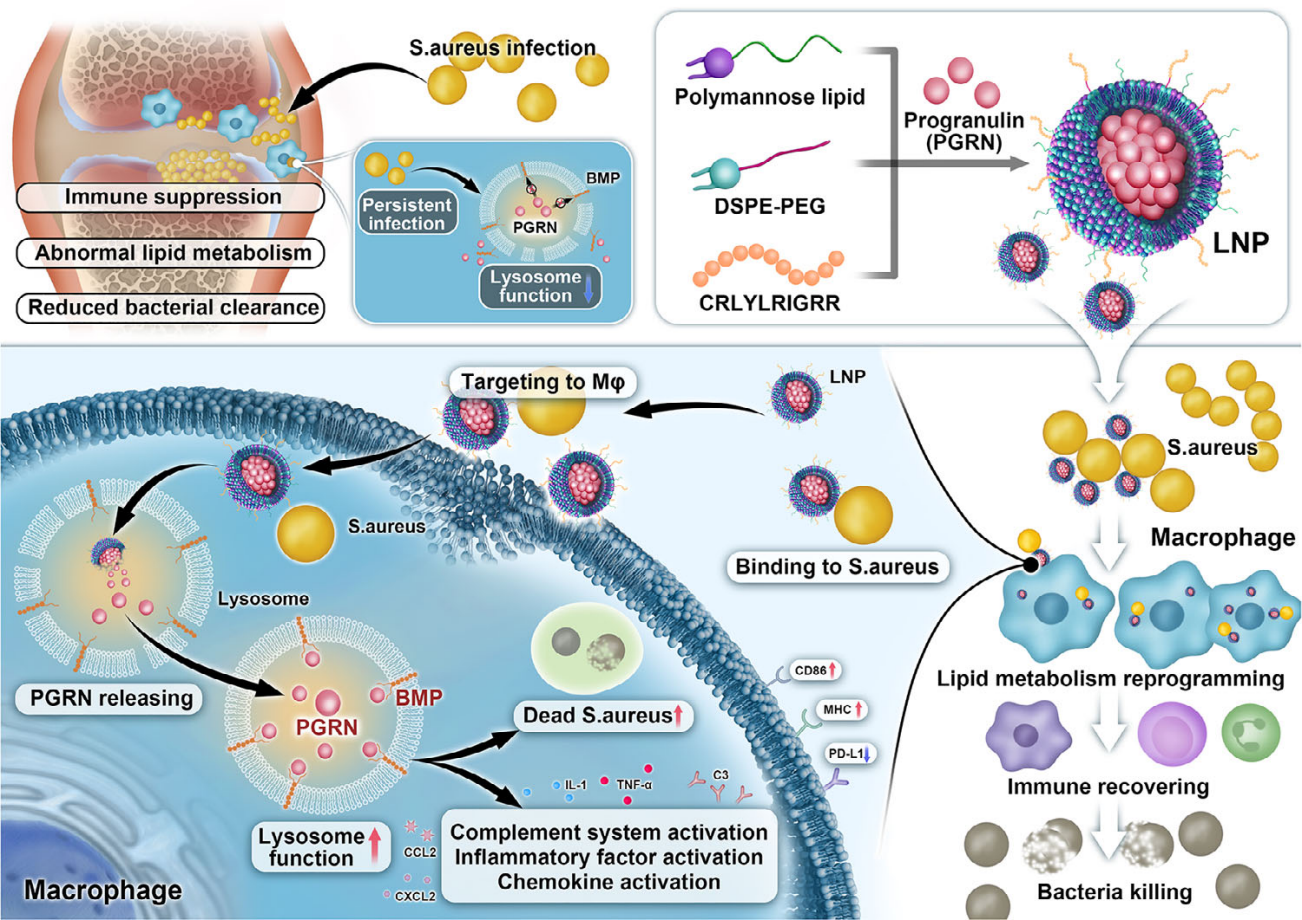
Prolonged intracellular bacterial infection induces macrophage lipid metabolic reprogramming, fostering an immunosuppressive microenvironment; Pathogen‐mediated dysregulation of PGRN expression impairs its binding to BMP‐a lipid critical for lysosomal membrane stability‐resulting in lysosomal dysfunction; Dual‐targeting nanoparticles restore lysosomal homeostasis through targeted PGRN delivery to lysosomes, reactivating pro‐inflammatory responses and remodeling the immunosuppressive microenvironment.

To elucidate this transition, we established a time‐course intracellular bacterial infection model in macrophages and conducted integrated transcriptomic and lipidomic profiling. Analyses revealed that prolonged intracellular infection induces macrophage lipid metabolic dysregulation, characterized by progressive accumulation of bis(monoacylglycero)phosphate (BMP). BMP is a glycerophospholipid primarily localized on the lysosomal membrane and involved in maintaining lysosomal membrane stability. Through its unique lipid structure, it enhances the physical stability and fluidity of the membrane, efficiently activates and recruits various lipid hydrolases (such as sphingomyelinase), thereby ensuring the proper degradation of complex lipids like cholesterol and sphingolipids [26, 27, 28]. Notably, lysosomal protein progranulin (PGRN) dynamically regulates BMP functionality through direct interaction [29, 30], which is critical for maintaining lysosomal homeostasis. PGRN deficiency exacerbates macrophage dysfunction and immunosuppression, facilitating bacterial immune evasion. In previous studies, PGRN has been extensively discussed as a multifunctional secreted glycoprotein with central roles in lysosomal homeostasis and the pathogenesis of aseptic arthritis. As a lysosomal chaperone, PGRN is transported into the lysosomal lumen where it binds to and stabilizes various lysosomal hydrolases such as β‐glucocerebrosidase, promoting their activity and ensuring normal lysosomal degradation [31, 32]; Moreover, PGRN specifically interacts with BMP on the lysosomal membrane, which is critical for regulating lysosomal lipid metabolism, membrane trafficking, and autophagic processes [29, 33]. Building on these findings, we propose targeting the “PGRN‐BMP‐lysosome” regulatory axis as a therapeutic strategy. Furthermore, we engineered a lysosome‐repairing nanomaterial designed to remodel the immunosuppressive microenvironment, synergistically enhancing antimicrobial efficacy and bone regeneration (Scheme 1).

## 2 Results

### 2.1 Sustained Intracellular Bacterial Persistence Impairs Macrophage Immune Function and Alters Lipid Metabolism

Once acute bacterial infections are not effectively cleared, they progress to persistent chronic infections. The transition is accompanied by remodeling of the local immune microenvironment, enabling intracellular pathogens to survive long‐term within macrophages [34, 35, 36, 37]. However, the mechanisms by which bacteria manipulate host immunity during the acute‐to‐chronic infection transition remain poorly characterized.

**FIGURE 1:**
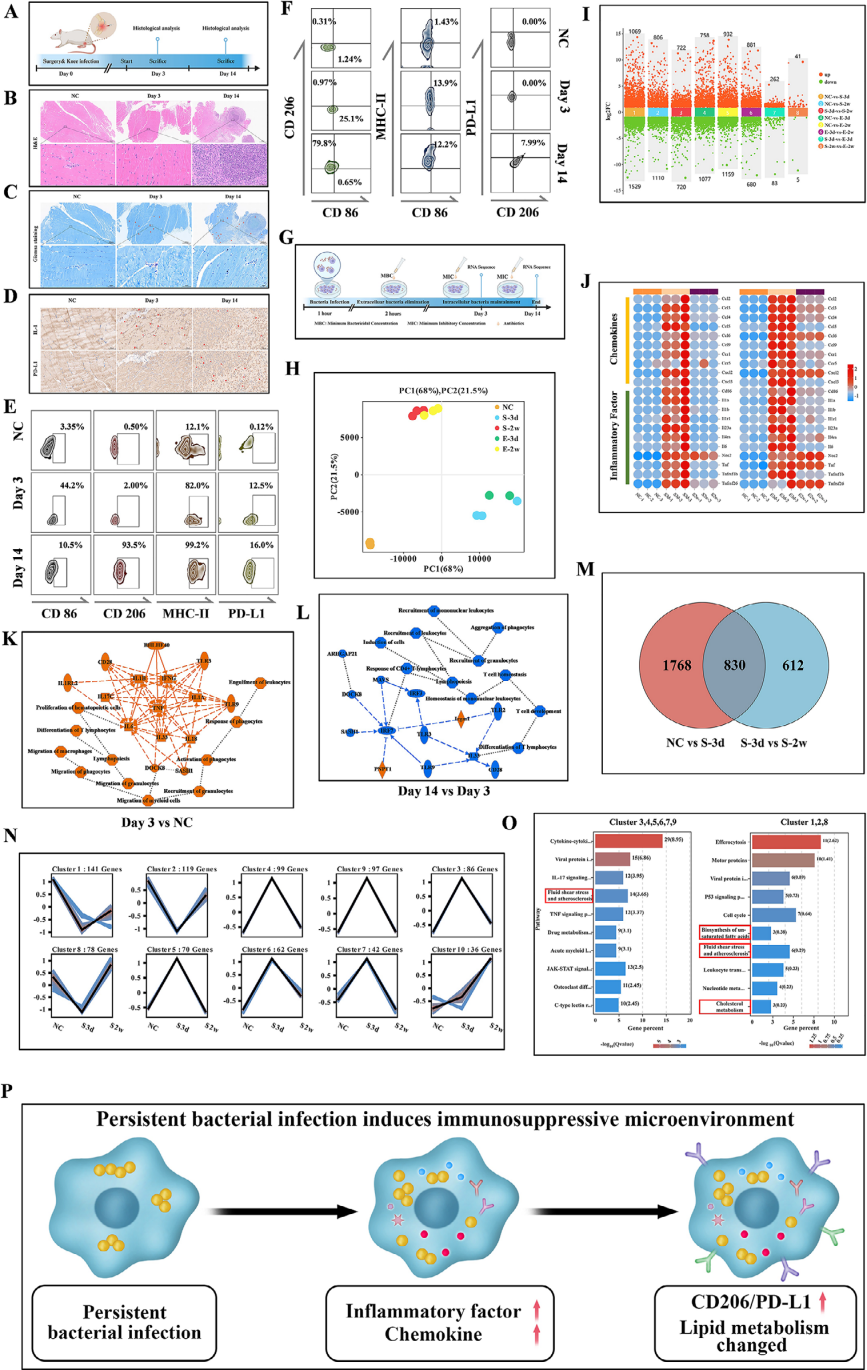
Local persistent infection leads to an altered immune status in the knee joint. (A) Schematic of the mouse knee joint infection model. (B) Representative H&E staining showing inflammatory infiltration in periarticular tissues over infection time (n = 3). (C) Representative Giemsa staining indicating infection severity (arrows denote bacterial presence) (n = 3). (D) Representative IHC depicting local immune response dynamics (n = 3). (E,F) Representative flow cytometry analysis of infected periarticular tissues (n = 3). (G) In vitro model of intracellular bacterial infection in macrophages for RNA‐seq analysis. (H) PCA of gene expression in different groups. (I) Multi‐group differential scatter plot of differentially expressed genes (DEGs) (n = 3). (J) Heatmap of inflammation‐related DEGs in *S. aureus*‐infected and *E. coli*‐infected groups (n = 3 per group). (K) IPA analysis of DEGs between *S. aureus* 3‐day infection and NC (negative control) groups (n = 3 per group). (L) IPA analysis of DEGs between S. aureus 14‐day and 3‐day infection groups (n = 3 per group). (M) Venn diagram of DEGs across different infection time points of *S. aureus* infected groups (n = 3 per group). (N) Trend analysis of 830 genes from (M), clustered into 10 patterns (n = 3 per group). (O) GO enrichment analysis of merged cluster genes from (N) (n = 3 per group). (P) Schematic of macrophage immune and metabolic changes during persistent infection.

To characterize time‐dependent changes in immune profiles, we established murine models of acute and chronic joint infections using *S. aureus*, the most prevalent pathogen in clinical bone and joint infections. Knee periarticular tissues were collected at Day 3 (acute phase) and Day 14 (chronic phase) post‐infection for histological evaluation and flow cytometric analysis (Figure 1A). As shown in Figure 1B,C and Figure S1A, bacterial loads progressively increased with prolonged infection duration (as indicated by red arrows in Giemsa staining), accompanied by gradual inflammatory cell infiltration. By Day 14, localized granuloma structures were observed. Further immunohistochemical (IHC) analysis revealed a biphasic trend in the expression of the immune activation marker IL‐1β, characterized by an initial increase followed by a decline during infection progression (Figure 1D; Figure S1B). In contrast, the immunosuppressive marker PD‐L1 exhibited a sustained upward trend (Figure 1D; Figure S1C).

Given the pivotal role of macrophages in immune responses, flow cytometric profiling was performed on periarticular tissue‐resident macrophages. Results demonstrated a predominant M1‐polarized phenotype (CD86**^+^**/CD206**^−^**) during the acute phase, which transitioned to an M2‐polarized state (CD86**^−^**/CD206**^+^**) in the chronic phase. Notably, a significant expansion of immunosuppressive macrophages (CD206**^+^**/PD‐L1**^+^**) was observed during the chronic phase (Figure 1E,F; Figure S1D–H).

Preliminary analysis of in vivo infected tissue samples confirmed the establishment of an immunosuppressive microenvironment by persistent bacterial infection. To further explore the impact of intracellular bacteria on macrophage functionality, we established in vitro infection models using RAW 264.7 macrophages infected with representative G+ (*S. aureus*) and G‐ (*E. coli*) pathogens, and performed transcriptomic sequencing (Figure 1G). PCA clustering analysis revealed that macrophages exhibited temporally conserved gene expression patterns during both G**^+^** and G^−^ bacterial infections. However, distinct transcriptomic divergence was observed between Day 3 and Day 14 post‐infection (Figure 1H). Sample correlation heatmap analysis further corroborated this temporal heterogeneity (Figure S1I). Comparative enrichment analysis of differentially expressed genes (DEGs) further corroborated this pattern, showing substantially greater gene count variations between timepoints than between bacterial species (Figure 1I). Notably, only 345 and 46 DEGs were identified according to bacteria category, respectively (Figure 1I, Groups 7 and 8), whereas 1442 and 1561 DEGs were detected, respectively, according to timepoints (Figure 1I, Groups 3 and 6).

Following bacterial invasion, Mφ rapidly activate immune responses through secretion of inflammatory cytokines and chemokines, whose intensity reflects the functional potency of the host immune system. Therefore, we performed enrichment analysis on the transcriptomic data focusing on inflammatory cytokines (IL‐1β, TNF‐α, etc.) and chemokines (CCL‐2, CCL‐3, etc.), which revealed significantly higher expression levels at Day 3 compared to Day 14 (Figure 1J; Figure S1J,K). To obtain a comprehensive landscape of immune dynamics following bacterial invasion, Ingenuity Pathway Analysis (IPA) was conducted. At Day 3, inflammatory pathways associated with granulocyte recruitment, leukocyte mobilization, and phagocyte activation were markedly upregulated, alongside immune mediators such as IL‐1, IL‐6, IFNG, and TNF. In contrast, by Day 14, inflammatory responses‐including granulocyte aggregation, lymphocyte recruitment, and neutrophil infiltration‐were suppressed, with concomitant reductions in associated cytokine expression (Figure 1K,L; Figure S1L,M).

Collectively, these in vivo results and in vitro transcriptomic data have indicated a macroscopic transition from immune activation to suppression following bacterial invasion. However, the underlying mechanisms driving this shift remained elusive. To investigate this transition, we identified intersecting DEGs across timepoints (Figure 1M; Figure S2A), yielding two gene subsets: 830 DEGs in the G^+^ group and 608 DEGs in the G^−^ group. Subsequent trend analysis of these DEGs generated 10 clusters (Figure 1N; Figure S2B), which broadly exhibited two distinct patterns: Pattern 1 showed transient upregulation (peaking at intermediate timepoints), whereas Pattern 2 demonstrated sustained upregulation over time.

KEGG pathway analysis of the two gene clusters yielded expected immune functional shifts in the top 10 enriched pathways, consistent with prior observations. Notably, both clusters revealed significant enrichment of lipid metabolism‐related pathways (Figure 1O; Figure S2C). In vitro observation of intracellular bacteria‐infected macrophages uncovered distinct morphological transitions: At Day 3, cells exhibited classical M1 features, including stellate morphology with extended pseudopodia and increased cytoplasmic granularity. By Day 14, macrophages adopted a rounded, enlarged morphology with pseudopodia retraction and prominent lipid droplet‐like structures (Figure S2D). IPA reaffirmed the profound impact of infection on macrophage lipid metabolism, with lipid‐related pathways ranking second only to immune pathways in the top 5 enriched pathways (Figure S2E,F). Strikingly, the top‐ranked liver X receptor (LXR) pathway‐critically linked to cholesterol metabolism, lipid homeostasis, and inflammatory regulation‐emerged as a central hub. This robust correlation between macrophage immunocompetence and lipid metabolic remodeling underscores a previously underappreciated mechanistic crosstalk.

### 2.2 Diverse Intracellular Bacterial Pathogens Converge on a Conserved Macrophage Lipid Metabolic Paradigm, Potentially Driving Immunosuppression via BMP Modulation

**FIGURE 2:**
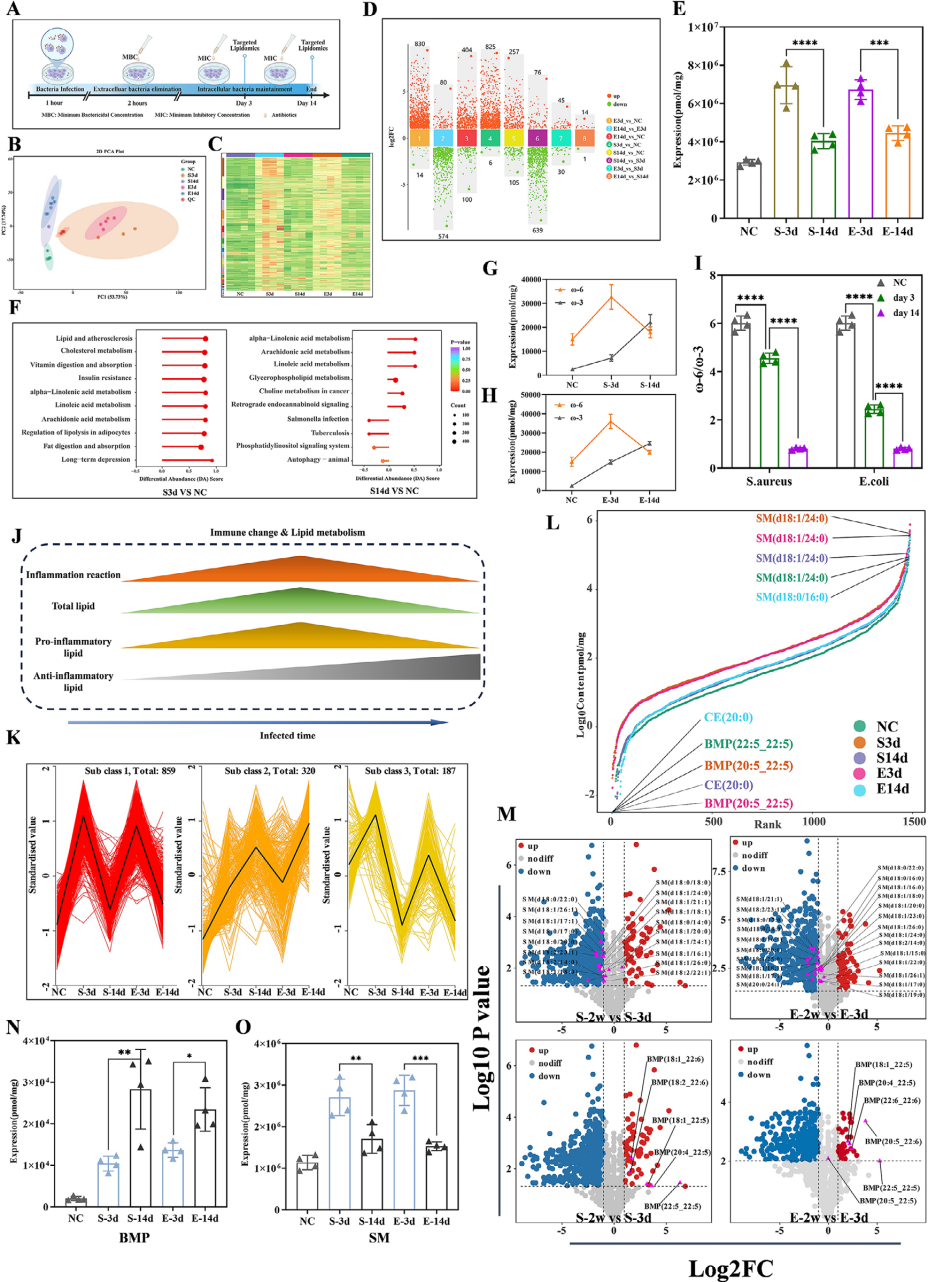
Lipid metabolism reprogramming the immune function of Mφ. (A) Schematic of the targeted lipidomics of intracellular bacteria infected Mφ (n = 4 per group). (B) PCA of lipids across different groups (QC: quality control) (n = 4 per group). (C) Heatmap of lipid expression profiles post‐infection (n = 4 per group). (D) Multi‐group differential scatter plot of significantly altered lipids (n = 4 per group). (E) Temporal changes in total lipid content of macrophages after bacterial infection (mean ± SD n = 4 per group). (F) GO enrichment analysis of differential lipids at distinct time points (*S. aureus* group shown) (n = 4 per group). (G–I) Temporal trends of ω‐3, ω‐6 fatty acids, and ω‐6/ω‐3 ratio post‐infection (mean ± SD n = 4 per group). (J) Schematic of immune function and lipid metabolic shifts during intracellular bacterial infection. (K) K‐means clustering analysis of lipid expression patterns across groups (n = 4 per group). (L) Dynamic distribution of lipid content in different groups (n = 4 per group). (M) Volcano plots of differentially regulated lipids (n = 4 per group). (N,O) Temporal trends of BMP and SM lipids during infection (n = 4 per group). Statistical significance was determined by one‐way ANOVA followed by Tukey's post hoc test. ^*^ *p* < 0.05; ^**^ *p* < 0.01; ^***^ *p* < 0.001; ^****^ *p* < 0.0001; ns, no significance.

Given the demonstrated impact of intracellular bacterial persistence on macrophage lipid metabolism, we proceeded to detailed lipidomic profiling. Mirroring the transcriptomic approach, intracellular bacterial infection models were established in RAW 264.7 macrophages, with targeted lipidomics performed at designated timepoints (Figure 2A). PCA analysis and hierarchical clustering heatmap of differential lipids revealed convergent temporal lipid metabolic patterns in macrophages infected with either G**^+^** or G**^−^** bacteria (Figure 2B,C). Comparative lipidomic profiling revealed only 15 differentially abundant lipids between *S. aureus*‐ and *E. coli*‐infected groups at Day 14 (Figure 2D, Group 8). In contrast, time‐dependent analyses within each group demonstrated hundreds of lipid species alterations when comparing Day 3 versus Day 14 outcomes (Figure 2D *S. aureus*: Group 6; *E. coli*: Group2). Quantitative analysis demonstrated dynamic lipid accumulation, characterized by an initial increase at Day 3 followed by significant depletion by Day 14, which paralleled the temporal trajectory of macrophage functional alterations (Figure 2E).

Lipid metabolites are intrinsically linked to immune functionality. KEGG analysis revealed significant temporal changes in inflammation‐associated lipid pathways such as arachidonic acid metabolism and linoleic acid metabolism at Day 3 and Day14 compared to the uninfected group (Figure 2F; Figure S2G). Under physiological conditions, ω‐6 fatty acids (e.g., linoleic acid LA) are metabolized to arachidonic acid (AA), a precursor of pro‐inflammatory mediators (prostaglandin E_2_, leukotriene B_4_, thromboxane A_2_) synthesized via cyclooxygenase (COX) and lipoxygenase (LOX) pathways. These mediators activate immune cells (e.g., macrophages, neutrophils), promoting vasodilation, chemokine release, and inflammatory cascades [38, 39, 40, 41]. Conversely, ω‐3 fatty acids (e.g., α‐linolenic acid, ALA) are converted to eicosapentaenoic acid (EPA) and docosahexaenoic acid (DHA), which generate anti‐inflammatory resolvins, protectins, and lipoxins [42, 43, 44]. These metabolites competitively inhibit AA metabolism to reduce pro‐inflammatory mediator production, promote inflammation resolution by suppressing neutrophil infiltration, enhance macrophage efferocytosis, and inhibit NLRP3 inflammasome activation to limit IL‐1β release [45, 46]. Lipidomic profiling demonstrated dynamic shifts in pro‐versus anti‐inflammatory lipid species: Pro‐inflammatory lipids (lysophosphatidic acid LPA, lysophosphatidylcholine LPC, platelet‐activating factor PAF) peaked at Day 3 before declining, whereas anti‐inflammatory fatty acids (FFA 20:5 EPA, FFA 22:6 DHA, ALA) progressively accumulated (Figure S2H,I). Concurrently, the ω‐6/ω‐3 fatty acid ratio exhibited a time‐dependent decline, driven by reduced ω‐6 levels and elevated ω‐3 abundance at Day 14 compared to Day 3 (Figure 2G–I). To delineate the functional roles of differential lipids, we performed intersectional analysis of time‐dependent lipid alterations in both *S. aureus*‐and *E. coli*‐infected groups, followed by pathway enrichment. While the top 10 pathways lacked direct immune‐related metabolic routes, both groups exhibited nearly identical pathway profiles (Figure S3C,D), reinforcing that macrophages adopt conserved lipid metabolic reprogramming patterns when challenged with divergent bacterial pathogens. This suggests a universal temporal lipid remodeling paradigm in macrophages during intracellular infection (Figure 2J). To systematically correlate lipid dynamics with immune functional states, all differentially abundant lipids across comparison groups were subjected to K‐means clustering analysis, yielding three distinct lipid clusters. Cluster 1 and 3 exhibited transient upregulation, while Cluster 2 demonstrated sustained accumulation over the infection course (Figure 2K). Subsequent pathway enrichment analysis of these clusters revealed striking polarization: anti‐inflammatory lipid metabolic pathways, efferocytosis‐related processes, and autophagy‐associated pathways were predominantly enriched in Clusters 2 (Figure S3A,B).

Functionally, macrophages mediate inflammation resolution and tissue repair through efferocytosis and autophagy. These findings suggest a dynamic equilibrium between pro‐ and anti‐inflammatory lipid metabolic programs throughout infection. Notably, anti‐inflammatory lipid metabolism ultimately prevailed during the chronic phase, dominating this molecular tug‐of‐war and directly contributing to macrophage functional suppression. To investigate how lipid metabolism modulates macrophage immune function, we focused on identifying critical lipid species by analyzing their dynamic abundance distribution ranges across experimental groups. This approach evaluates the highest‐ and lowest‐abundance lipids and tracks temporal shifts in lipid abundance spans. Results demonstrated that sphingomyelin (SM) consistently ranked as the most abundant lipid across all groups. Strikingly, the lowest‐abundance lipids exhibited phase‐dependent divergence: At Day 3, BMP showed minimal abundance in both *S. aureus*‐ and *E. coli*‐infected groups, mirroring uninfected controls (NC group). By Day 14, cholesterol esters (CEs) became the least abundant species in infected groups (Figure 2L). This temporal inversion in low‐abundance lipid profiles indicates infection duration‐dependent metabolic remodeling. Volcano plot analysis revealed significant differential abundance of the lowest‐abundance lipid (BMP) and highest‐abundance lipid (SM) across all timepoints (Figure 2M). Further temporal profiling showed that SM exhibited a biphasic trend‐initial elevation followed by decline‐whereas BMP demonstrated progressive accumulation throughout infection (Figure 2N,O). Notably, among all lipid subclasses analyzed, BMP was the sole species showing sustained upregulation in both *S. aureus*‐ and *E. coli*‐infected macrophages over time. Given the continuous molecular competition between pro‐ and anti‐inflammatory forces from infection onset, we hypothesize that BMP accumulation may serve as a mechanistic driver of immunosuppression during chronic infection. Its gradual enrichment likely tilts the immunometabolic balance toward anti‐inflammatory dominance, ultimately compromising macrophage antimicrobial functions in later stages.

### 2.3 Sustained Intracellular Bacterial Persistence Impairs Macrophage Immune Function via PGRN‐BMP‐Lysosome Axis

BMP, a unique glycerophospholipid, exhibits structural and functional differences from canonical phospholipids. Its glycerol backbone contains two hydroxyl groups each esterified with a single fatty acyl chain (monoacylation), while the third hydroxyl forms a phosphodiester bond. Despite variability in fatty acid chain length/saturation, BMP demonstrates strict subcellular localization, predominantly enriched in lysosomes and late endosomes. As a critical lysosomal membrane component, BMP facilitates acid lipase‐mediated degradation of complex lipids (e.g., sphingolipids, cholesterol esters) by stabilizing specialized membrane microdomains, and is pathologically linked to lysosomal storage disorders such as Niemann‐Pick disease type C [47, 48]. Unlike common phospholipids (e.g., phosphatidylcholine), BMP's monoacylated structure and lysosomal tropism render it uniquely specialized in lipid catabolism and membrane remodeling. Clinically, aberrant BMP accumulation serves as a biomarker for lysosomal dysfunction and neurodegenerative diseases [49, 50, 51].

**FIGURE 3:**
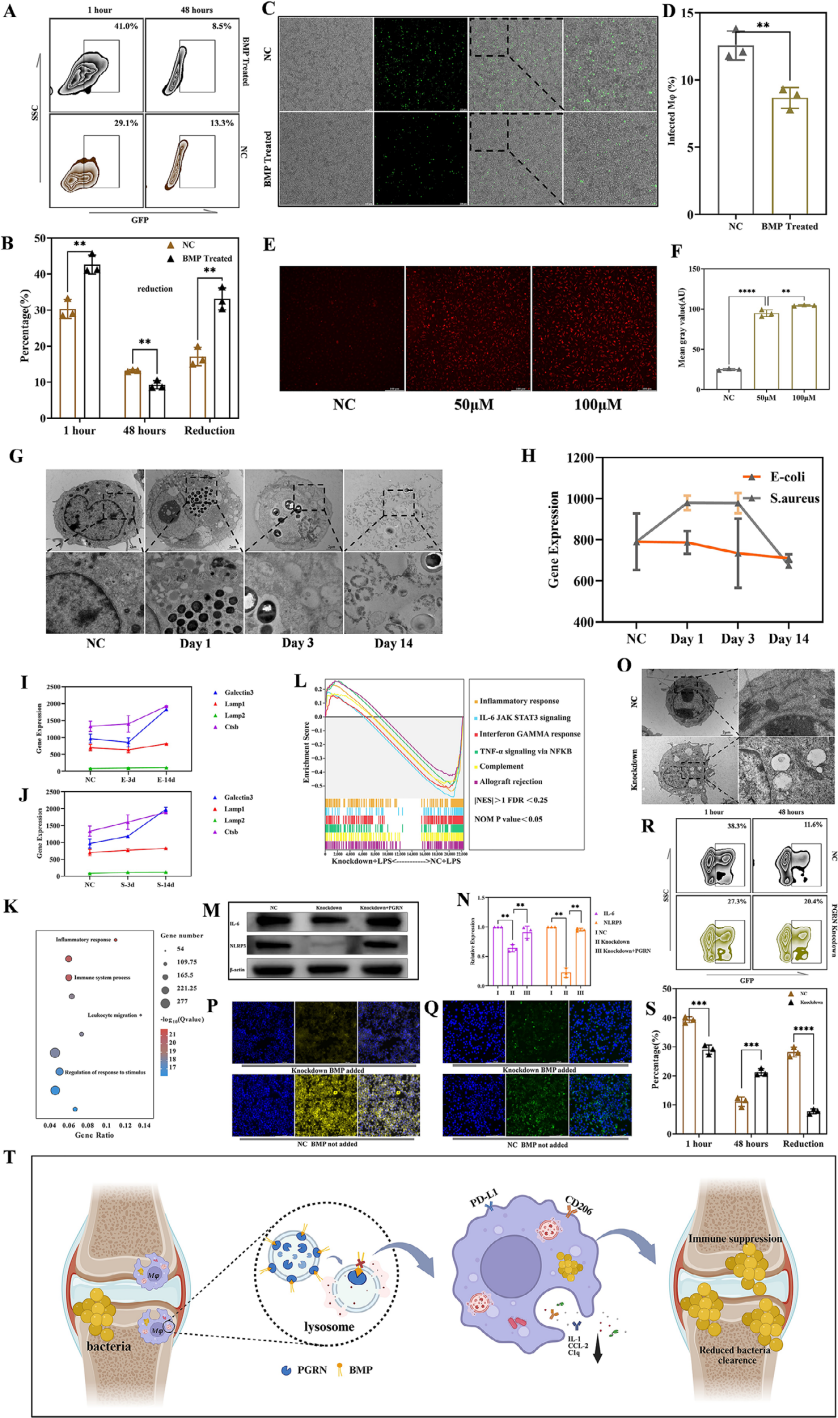
BMP regulates macrophage antibacterial functions through PGRN‐mediated lysosomal modulation. (A,B) Flow cytometry analysis showing BMP pretreatment enhances bacterial phagocytosis by macrophages (GFP signal represents *MRSA‐USA300*, mean ± SD n = 3). (C,D) BMP post‐treatment improves intracellular bacterial clearance in macrophages (green signal represents *MRSA‐USA300*, mean ± SD n = 3). (E,F) BMP increases lysosomal quantity and function in macrophages (lysosome were marked by lysotracker‐red, mean ± SD n = 3). (G) Representative TEM images showing lysosomal morphological changes in macrophages at different infection time points. (H) Temporal expression pattern of PGRN in macrophages during infection (mean ± SD n = 3). (I,J) Expression trends of lysosomal damage‐related genes during infection in *E. coli* and *MRSA* group, respectively (mean ± SD n = 3). (K) GO enrichment of DEGs in macrophages under LPS stimulation (PGRN‐knockdown vs NC, both of two groups were treated with LPS). (L) GSEA analysis of differential genes between PGRN‐knockdown and NC groups (both of two groups were treated with LPS). (M,N) Western blot analysis of inflammatory markers in PGRN‐knockdown vs normal macrophages under LPS stimulation (mean ± SD n = 3). (O) Comparison of lysosomes between PGRN‐knockdown and normal macrophages under TEM. (P,Q) Rescue effect of BMP treatment on lysosomal function in PGRN‐knockdown macrophages ((P) lysotracker; (Q) DQ‐BSA). (R,S) Impaired antibacterial capacity in PGRN‐knockdown macrophages (mean ± SD n = 3). (T) Schematic of how intracellular bacteria subvert immune response via PGRN‐BMP axis‐mediated lysosomal dysfunction. Comparisons involving three or more groups were analyzed by one‐way ANOVA with Tukey's post hoc test. Unpaired Student's t‐tests were used for comparisons between two independent groups. ^*^ *p* < 0.05; ^**^ *p* < 0.01; ^***^ *p* < 0.001; ^****^ *p* < 0.0001; ns, no significance.

To investigate its role in macrophage bactericidal capacity, macrophages pre‐treated with BMP for 12 h were compared with untreated controls in bacterial phagocytosis assays. Paradoxically, flow cytometry revealed enhanced phagocytic uptake of GFP‐expressing bacteria in BMP‐treated macrophages at 1 h post‐infection, with significantly reduced intracellular bacterial loads at 48 h (Figure 3A,B). Subsequently, we reversed the treatment sequence: macrophages were first infected with intracellular bacteria, followed by BMP administration. Consistent with prior findings, BMP‐treated macrophages exhibited superior bacterial clearance compared to untreated controls at 48 h (Figure 3C,D). These results demonstrate that BMP enhances macrophage antimicrobial function regardless of treatment timing. Furthermore, BMP treatment increased lysosomal abundance and acidification (Figure 3E,F). These findings starkly contradict our initial hypothesis that BMP accumulation drives immunosuppression. This paradox suggests regulatory mechanisms that compromise BMP functionality during chronic infection, despite its quantitative enrichment. We then revisited the relevant literature and discovered that BMP is primarily localized to the lysosomal membrane, where it binds to howand contributes to maintaining lysosomal membrane stability [26, 47, 52]. And studies indicate that progranulin (PGRN), after synthesis, is trafficked to lysosomes where it interacts with BMP to maintain lysosomal membrane integrity [29, 53]. Besides, recent studies on neurological disorders implicating PGRN in lysosomal homeostasis prompted us to explore its potential role in this regulatory axis [53, 54, 55, 56, 57]. We systematically assessed lysosomal functionality in intracellular bacteria‐infected macrophages. Transmission electron microscopy (TEM) revealed intact lysosomal morphology during early infection (Day 3), whereas by Day 14, lysosomal numbers increased but exhibited compromised membrane integrity, accompanied by global cellular membrane destabilization (Figure 3G). Temporal profiling of PGRN expression demonstrated an initial elevation followed by significant decline in S. aureus‐infected macrophages, while *E. coli*‐infected cells showed stable early‐phase expression followed by late‐phase suppression (Figure 3H). Despite pathogen‐specific kinetic differences, both groups exhibited marked PGRN downregulation by Day 14. Concurrently, lysosomal damage‐associated genes showed progressive upregulation in both infection models (Figure 3I,J). These findings collectively suggest that PGRN acts as a functional switch governing BMP‐mediated lysosomal homeostasis, thereby modulating immune outcomes.

To validate this hypothesis, we generated stable PGRN‐knockdown macrophages using shRNA. Upon lipopolysaccharide (LPS) stimulation, qPCR revealed significantly reduced expression of pro‐inflammatory cytokines (IL‐1β), complement components (C1q), chemokines (CCL2), and M1 polarization markers (CD86) in PGRN‐deficient cells (Figure S4A). RNA‐seq confirmed profound immunophenotypic divergence: PCA showed clear separation between NC and shRNA groups (Figure S4B), with the top 5 DEGs enriched for immune activation pathways in NC group versus anti‐inflammatory/repair pathways in shRNA groups (Figure S4C). GO analysis identified four immune‐related pathways among the top 10 terms (Figure 3K), while gene set enrichment analysis (GSEA) demonstrated attenuated activation of inflammatory pathways in PGRN‐deficient macrophages post‐LPS stimulation (Figure 3L). Western blotting corroborated these findings, showing suppressed IL‐1β and NLRP3 protein levels in knockdown groups (Figure 3M,N). Furthermore, TEM revealed pathological lysosomal enlargement with reduced electron density in PGRN‐deficient macrophages, indicative of lysosomal dysfunction (Figure 3O). Lysosomal labeling using LysoTracker and functional test using DQ‐BSA assays demonstrated that exogenous BMP supplementation failed to restore lysosomal functionality in PGRN‐deficient macrophages (Figure 3P,Q). This defect ultimately manifested as impaired bacterial phagocytosis and diminished intracellular bacterial clearance capacity, which was quantitatively confirmed by flow cytometric analysis (Figure 3R,S).

The functional integrity of macrophage lysosomes serves as a critical nexus bridging innate and adaptive immunity, essential for comprehensive immune system activation. Following pathogen phagocytosis, lysosomes employ acidic hydrolases to degrade bacterial antigens into small peptide fragments. These fragments are subsequently loaded onto major histocompatibility complex class II (MHC‐II) molecules within lysosomal compartments, forming antigen‐MHC‐II complexes that translocate to the cell surface for antigen presentation. This process directly activates CD4^+^ T cells (helper T cells), initiating antigen‐specific humoral and cellular immune responses. Furthermore, lysosomes regulate inflammasome activation and cytokine release (e.g., IL‐1β, IL‐6), amplifying local inflammatory reactions and immune cell recruitment.

Impaired lysosomal enzymatic activity or aberrant antigen processing diminishes antigen presentation efficiency, attenuates T cell activation, and may ultimately induce immune tolerance or chronic infection. Crucially, the PGRN‐BMP complex is indispensable for maintaining lysosomal membrane stability. Persistent intracellular bacteria downregulate PGRN expression in macrophages, destabilizing lysosomal membranes and impairing their functionality. This lysosomal dysfunction suppresses inflammatory signaling and facilitates bacterial immune evasion (Figure 3T).

### 2.4 Development of NPs@PGRN and Its Characterization

**FIGURE 4:**
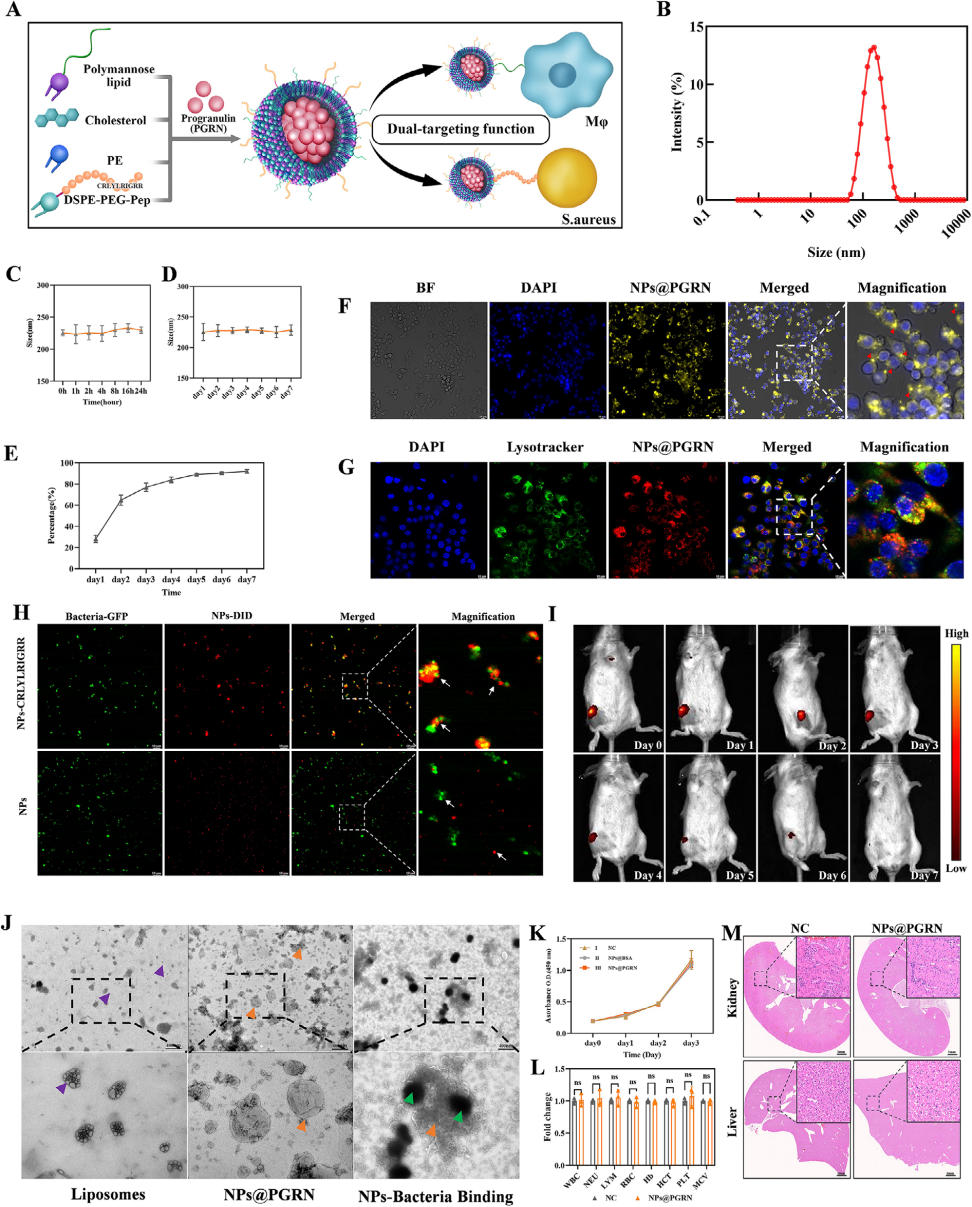
Synthesis and characterization of nanoparticles. (A) Synthesis scheme of NPs@PGRN nanoparticles. (B) DLS analysis of nanoparticles. (C) Particle size variation trend of the nanomaterial in 10% FBS DMEM solution at 37°C over 24 h (mean ± SD n = 3); (D) Particle size variation trend of the nanomaterial in 10% FBS DMEM solution at 4°C over 1 week (mean ± SD n = 3); (E) Release profile of the nanomaterial in PBS at 37°C over 1 week (mean ± SD n = 3); (F) Process of nanoparticle phagocytosis by macrophages (red arrows: NPs@PGRN). (G) Confocal fluorescence microscopy showing nanoparticle uptake and subsequent targeting to lysosomes. (H) Specific binding of the nanomaterial to bacteria (red: DID‐labeled nanomaterials; green: *MRSA‐GFP*) (I) In vivo fluorescence imaging demonstrating the release process of NPs@PGRN (n = 3); (J) TEM showing nanoparticle morphology and nanoparticle‐bacteria binding (purple arrow: liposomes; yellow arrow: NPs@PGRN; green arrow: *MRSA*). (K) CCK‐8 assay investigating the effect of NPs@PGRN on the proliferation of primary chondrocytes (n = 5). (L) Hematotoxicity assessment of NPs@PGRN (mean ±SD n = 3); (M) Hepatorenal toxicity evaluation of NPs@PGRN (n = 3). Comparisons involving three or more groups were analyzed by one‐way ANOVA with Tukey's post hoc test. Unpaired Student's t‐tests were used for comparisons between two independent groups. ^*^ *p* < 0.05; ^**^ *p* < 0.01; ^***^ *p* < 0.001; ^****^ *p* < 0.0001; ns, no significance.

Intracellular bacteria exploit the PGRN‐BMP‐lysosome axis to destabilize lysosomal membranes, enabling chronic parasitism. To counteract this, we aimed to restore lysosomal integrity and reactivate macrophage inflammatory responses. Given the infection‐driven BMP accumulation, we hypothesized that supplementing deficient PGRN could rescue lysosomal function. This requires macrophage‐specific targeting and efficient PGRN delivery. To achieve this, we engineered a bioresponsive nanoparticle (NP) system for precise macrophage immunomodulation. Since molecules or nanoparticles bearing mannosyl groups can be internalized by macrophages via mannose receptors, we first synthesized mannose‐bearing polymers and successfully obtained the target product‐polymannose (PM30), and the formation of PM30 was primarily verified by ^1^H‐NMR analysis (Figures S4D and S5A). Furthermore, to more effectively eliminate free bacteria that have escaped into the cytosol as well as residual extracellular bacteria, a bacteria‐targeting peptide (CRLYLRIGRR) was designed and synthesized according to the previous study [58, 59] (Figure S5B,C) and incorporated the targeting peptide into the nanoliposomes using DSPE‐PEG (Figure S6A,B). This dual‐functional system enables NPs to first capture bacteria, then deliver them into macrophages for lysosomal elimination. The final NPs@PGRN liposomal nanocomplex was assembled via stepwise integration of these components (Figure 4A). Dynamic light scattering (DLS) demonstrated uniform NPs size distribution (∼200 nm, Figure 4B), while TEM revealed enhanced morphological homogeneity compared to plain liposomes (Figure 4J). Since the molecular weight of the PGRN protein is similar to that of BSA, we used BSA as a substitute for PGRN to perform encapsulation efficiency testing and release kinetics analysis of the nanoparticle system. The encapsulation efficiency for PGRN, as determined by the Bradford assay, ranged from 20% to 30%. To test the stability of the nanomaterials in biological fluids, the prepared liposomes were uniformly dispersed in DMEM medium containing 10% FBS and incubated in a cell culture incubator at 37°C with 5% CO_2_. DLS measurements were performed at various time points within a 0–24 h time‐frame to simulate the stability of the nanoparticles in biological fluids. The results showed that over the 24‐h period, the nanomaterials maintained a stable particle size of approximately 200 nm (Figure 4C). Given that liposomes are not suitable for freezing, and in consideration of the long‐term storage stability of the prepared liposomes, we also evaluated their stability in DMEM medium containing 10% FBS at 4°C over a 7‐day period. The results indicated that on the 7th day, the liposomes still maintained good stability (Figure 4D). Release kinetics assay revealed that within the first 24 h, approximately 20%–30% of the protein was released; by 72 h, the release reached 60%–70%, and after 1 week, approximately 90% of the protein had been released (Figure 4E).

To investigate the potential off‐target effects, we conducted exploratory experiments using both conventional fluorescence microscopy and confocal microscopy. First, we labeled the PGRN protein with the Cy5 fluorescent dye and subsequently synthesized NPs@PGRN‐Cy5, which incorporated the Cy5‐labeled PGRN. After incubating NPs@PGRN‐Cy5 with macrophages for 30 min, fluorescence imaging revealed efficient NPs internalization by macrophages, ensuring effective intracellular PGRN delivery to the cells (Figure 4F, as indicated by red arrows). To clarify the intracellular delivery efficiency of PGRN by the nanoparticles, we extracted intracellular proteins from day 0 to day 5 for Western blot analysis (Figure S7C,D). The results indicated that intracellular PGRN levels remained elevated during the first three days post‐delivery, began to decline on day 4, but still remained higher than in the non‐delivered group (day 0).

Furthermore, we pre‐labeled the macrophage lysosomes with Lysotracker, and then incubated the labeled macrophages with the nanoparticles. Confocal microscopy revealed extensive colocalization of NPs@PGRN‐Cy5 with lysosome, indicating that the nanoparticles were indeed taken up into the lysosomal compartments of the macrophages (Figure 4G). Besides, time‐course analysis of PGRN colocalization with lysosomes revealed strong colocalization of the PGRN Cy5 signal with lysosomal markers, and the fluorescence intensity remained high during the first three days, consistent with the Western blot data (Figure S7A,B).

To elucidate the specific process by which PGRN nanoliposomes deliver PGRN to lysosomes for binding with BMP, thereby restoring lysosomal function to exert therapeutic effects, we adopted a bidirectional validation strategy. First, we pretreated PGRN knockdown macrophages with 300 nm PLA2G15 (a BMP hydrolase that specifically degrades BMP) for 12 h to hydrolyze intracellular BMP lipids, followed by delivery of NPs@PGRN. Assessment of CTSD hydrolase activity and lysosomal membrane permeability showed that, compared with macrophages not treated with the hydrolase, cells receiving PLA2G15 pretreatment failed to restore lysosomal function or lysosomal membrane stability even after PGRN supplementation. The intracellular bacterial killing capacity was likewise not improved (Figure S7E).

Next, treatment of macrophages with bafilomycin A1 to block the fusion of nanoliposomes with lysosomes demonstrated that when PGRN was delivered via the nanoliposomes, the blockade by bafilomycin prevented PGRN from improving lysosomal function or membrane stability, and no enhancement in bactericidal activity was observed (Figure S7F). These results support the conclusion that NPs@PGRN restore lysosomal function primarily by delivering the PGRN protein directly to the lysosomal membrane.

To assess the targeting capability of nanosystem against *MRSA*, NPs (without CRLYLRIGRR) and NPs‐ CRLYLRIGRR (with CRLYLRIGRR) were first labeled with the DID dye and then incubated with *MRSA‐USA300‐GFP* for 10 min, respectively. Results are visualized using fluorescence confocal microscopy. The red fluorescence represents liposomes labeled with the DID dye, and the green fluorescence indicates bacteria. Notably, in the NPs‐CRLYLRIGRR group, the red fluorescence is clearly clustered and shows significantly increased intensity. This aggregation is due to the targeting effect of the antimicrobial peptide on the bacteria, leading to the accumulation of nanoparticles around the bacterial cells. In contrast, the control group (without CRLYLRIGRR) shows a diffuse and non‐specific distribution. The merged images further demonstrate close colocalization between the liposomes containing the antimicrobial peptide (red) and the bacteria (green), indicating tight binding. In the control group, no obvious binding is observed between the two, confirming that the antimicrobial peptide exhibits effective bacterial targeting capability (Figure 4H). And TEM results also showed dense NPs‐ CRLYLRIGRR aggregation around *MRSA* within 10 min of co‐incubation, confirming the bacterial targeting (Figure 4J).

To evaluate the biodistribution of the nanodrug, 50 µL of NPs@PGRN‐Cy5(PGRN:1 µg/mL) nanoparticles were injected into the knee joints of mice. Subsequent in vivo fluorescence imaging showed that the fluorescent signal persisted for up to 1 week, indicating that the nanomaterials exhibited a certain degree of tissue retention and likely possessed a prolonged sustained‐release effect (Figure 4I). Notably, we did not observe significant enrichment of the fluorescent signal in the heart or liver. We believe this may be related to the mode of local administration in the knee joint. Unlike conventional intravenous administration of nanomaterials, the knee joint has limited vascularization, resulting in very slow entry of nanoparticles into the systemic circulation. As a result, only a small number of nanoparticles enter the bloodstream per unit time, and the corresponding fluorescence signal is insufficient to produce detectable enrichment in the liver or kidneys. This interpretation is further supported by the prolonged signal persistence compared to that typically seen after intravenous injection. This prolonged signal decay period further suggests a slower metabolism and extended retention time at the local injection site.

To assess the biosafety​ of NPs@PGRN, endotoxin testing was performed by limulus amebocyte lysate (LAL) chromogenic assay. The final results showed that the endotoxin level was below 0.01 EU/mL. Considering that PGRN, as a growth factor‐like protein, may stimulate the proliferation of surrounding tissue cells and induce adverse reactions, we performed CCK‐8 assays using primary mouse chondrocytes in vitro.​ Results indicated no significant proliferative effect of the treatment compared to the control (Figure 4K).

Although in this study the administration route was local injection into the knee joint, assessing the systemic toxicity of the nanomaterials remains critically important. 50 µL of NPs@PGRN‐Cy5 (PGRN:1 µg/mL) nanoparticles were injected into the knee joints of healthy mice. Fourteen days after injection, the mice were euthanized, and liver and kidney tissues were collected for histological analysis using hematoxylin and eosin (HE) staining. The results showed no obvious tissue damage in the liver or kidneys of the NPs@PGRN‐injected group compared to the normal control group (Figure 4M). In addition, routine blood tests performed 14 days after injection revealed no significant differences between the two groups in standard parameters such as red blood cells, white blood cells, and hemoglobin levels (Figure 4L).

### 2.5 Therapeutic Efficacy of NPs@PGRN In Vitro

**FIGURE 5:**
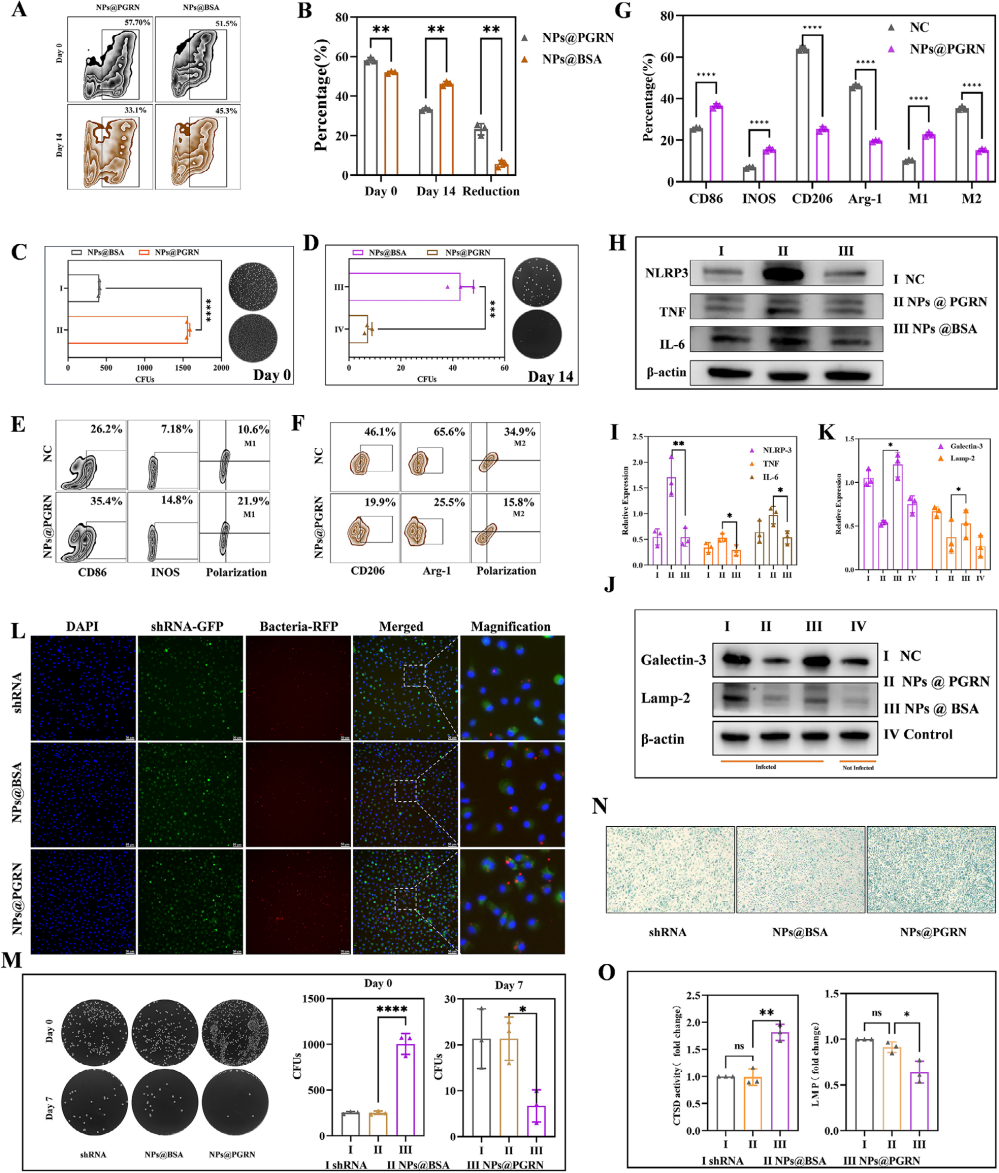
In vitro effects of NPs@PGRN on macrophage function. (A,B) NPs@PGRN enhance antibacterial capacity in PGRN‐knockdown macrophages (RAW264.7) in vitro (mean ± SD n = 3). (C,D) Bacterial colony formation assay of PGRN‐knockdown macrophages after nanoparticle treatment (mean ± SD n = 3). (E–G) NPs@PGRN treatment restores immune function in macrophages at 14 days post‐infection (14‐day infection followed by 24‐h treatment, mean ± SD n = 3). (H,I) Western blot analysis of inflammatory mediators in 14‐day infected macrophages after nanoparticle treatment (RAW264.7, 14‐day infection followed by 24‐h treatment, mean ± SD n = 3). (J,K) Western blot analysis of lysosomal damage‐related proteins in 14‐day infected macrophages post nanoparticle treatment (RAW264.7, 14‐day infection followed by 24‐h treatment, mean ± SD n = 3). (L,M) In PGRN‐knockdown primary bone marrow‐derived macrophages (BMDMs), NPs@PGRN enhanced bacterial phagocytosis and reduced intracellular bacterial survival; (N) In PGRN‐knockdown BMDMs, NPs@PGRN treatment increased β‐galactosidase activity (n = 3). (O) In PGRN‐knockdown primary BMDMs, NPs@PGRN treatment enhanced cathepsin D (CTSD) enzyme activity and reduced lysosomal membrane permeability (LMP) (mean ± SD n = 3). Comparisons involving three or more groups were analyzed by one‐way ANOVA with Tukey's post hoc test. Unpaired Student's t‐tests were used for comparisons between two independent groups. ^*^ *p* < 0.05; ^**^ *p* < 0.01; ^***^ *p* < 0.001; ^****^ *p* < 0.0001; ns, no significance.

To validate the in vitro antimicrobial efficacy of the synthesized nanoparticle, PGRN‐knockdown RAW264.7 cells were employed to mimic the macrophages under chronic infection state. Flow cytometry demonstrated that NPs@PGRN‐treated macrophages exhibited significantly enhanced bacterial phagocytosis at Day 0 compared to untreated controls, indicating partial restoration of phagocytic capacity. By Day 14, NPs@PGRN‐treated macrophages showed markedly reduced intracellular bacterial retention and accelerated bacterial clearance kinetics relative to controls, confirming that NPs@PGRN effectively restored the bactericidal function of lysosome‐impaired macrophages (Figure 5A,B). These findings were further corroborated by colony‐forming unit (CFU) assays (Figure 5C,D).

M2 polarization of macrophages during chronic infection is a critical contributor to bacterial immune evasion. Reverting macrophages to an M1‐polarized state is essential for bacterial eradication. Thus, NPs@PGRN treatment was administered at Day 14 post‐infection. Normal RAW264.7 cells were employed in both groups. NPs@PGRN treatment significantly reduced expression of the M2 markers CD206 and Arg‐1, while elevating levels of the M1 markers CD86 and inducible nitric oxide synthase (iNOS), and it decreased the proportion of immunosuppressive M2 macrophages compared to controls (Figure 5E–G). Concurrently, NPs@PGRN robustly reactivated pro‐inflammatory responses (Figure 5H,I). Western blot analysis of lysosomal damage‐associated proteins confirmed that NPs@PGRN effectively repaired intracellular bacteria‐induced lysosomal dysfunction (Figure 5J,K).

Next, we validated the effects of NPs@PGRN in mouse bone marrow‐derived macrophages (BMDMs). We employed AAV viruses to knock down PGRN expression in BMDMs. Since GFP was used as the marker protein for viral transfection, the fluorescence signal overlapped with that of *MRSA‐USA300‐GFP*, making it impossible to distinguish between the two. Therefore, we constructed a strain of *MRSA‐USA300‐RFP* expressing a red fluorescent protein marker. This RFP‐expressing strain emits ​​red fluorescence under Cy5 channel excitation​​ after treatment with ​​biliverdin hydrochloride. For the infection assay, an ​​MOI (multiplicity of infection) of 50:1​​ was used, and the bacteria and cells were co‐incubated for ​​30 min​​. After incubation, the cells were washed twice with ​​PBS to remove unattached bacteria​​, and then observed under a ​​fluorescence microscope. The cells were lysed on Day 0 and Day 7 post‐infection for CFU analysis.

The results showed that compared to the blank group (shRNA) and the control group (NPs@BSA), BMDMs treated with NPs@PGRN exhibited enhanced phagocytic capacity, as evidenced by an increased number of red fluorescent signals (Figure 5L). This observation was further confirmed by the CFU assay. By day 7, the NPs@PGRN group exhibited significantly fewer bacterial residues compared to the other two groups (Figure 5M).

In terms of lysosomal function in the treated macrophages, lysosomal ​​β‐galactosidase staining​​ revealed deeper staining in the NPs@PGRN group (Figure 5N). Furthermore, the NPs@PGRN group demonstrated higher ​​cathepsin D (CTSD) enzymatic activity​​ in lysosomes and lower ​​lysosomal membrane permeability (LMP) (Figure 5O).

In vitro findings demonstrate that nanomaterial‐mediated delivery of PGRN to lysosomal compartments reactivates macrophage pro‐inflammatory responses and facilitates the clearance of chronically persisting intracellular pathogens.

### 2.6 NPs@PGRN Modulates In Vivo Immune Landscapes and Reduces Bacterial Burden

**FIGURE 6:**
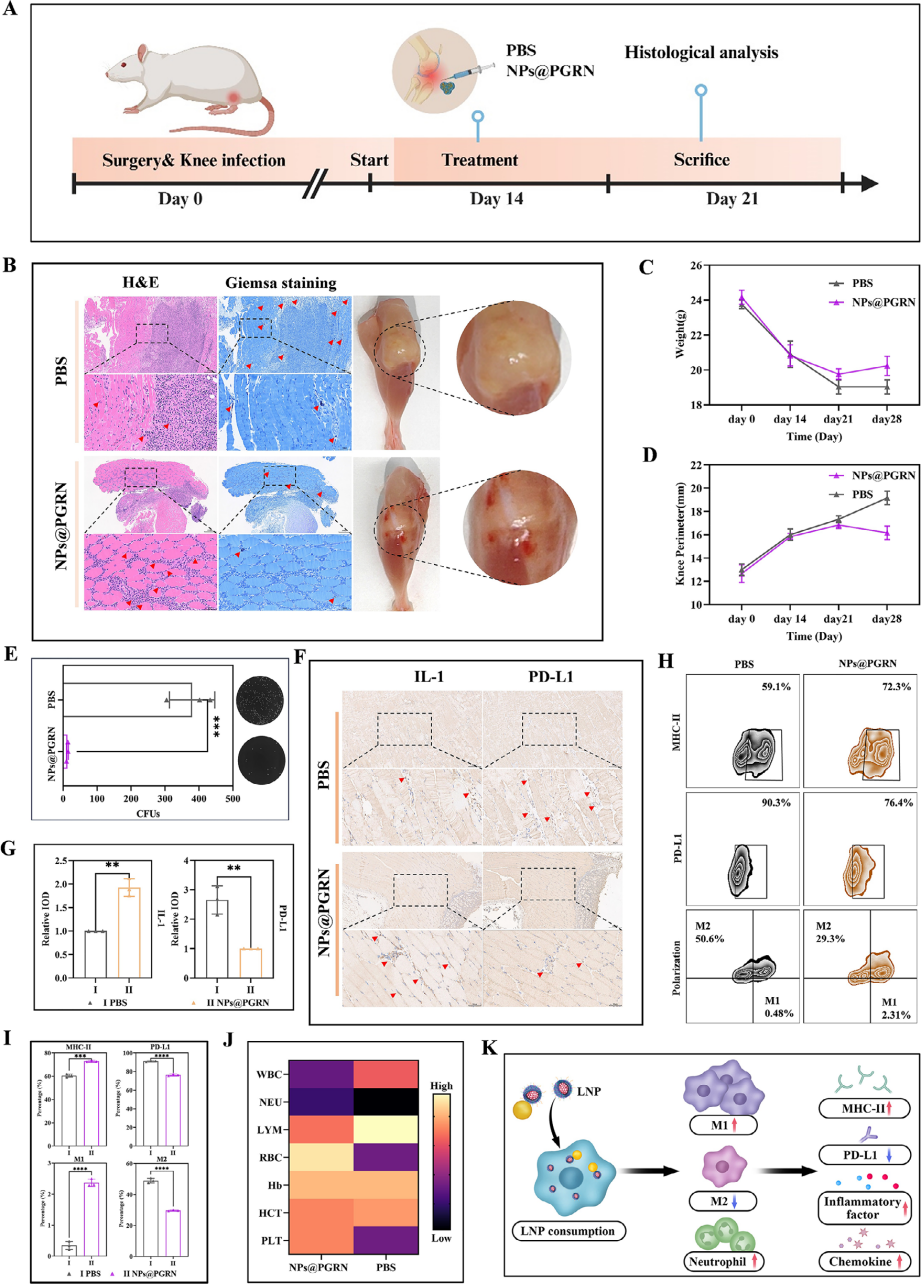
Therapeutic effects of NPs@PGRN in vivo. (A) Schematic of the nanoparticle treatment protocol in vivo. (B) H&E and Giemsa staining of periarticular tissues (H&E: arrows indicate neutrophil infiltration; Giemsa staining: arrows indicate bacteria, n = 3 per group). (C) Body weight changes of mice during treatment. (D) Changes in knee joint circumference of affected limbs (mean ± SD n = 3). (E) Bacterial colony formation assay from intracellular bacteria in periarticular macrophages (mean ± SD n = 3). (F) IHC staining of immune markers in periarticular soft tissues (red arrows indicate positive staining). (G) Quantitative analysis of IL‐1 and PD‐L1 expression from IHC staining in Figure 6F (mean ± SD n = 3). (H,I) Flow cytometry analysis of immune markers in knee joint macrophages (mean ± SD n = 3). (J) Complete blood count (CBC) analysis. (K) Schematic summarizing the in vivo therapeutic mechanism of NPs@PGRN. Comparisons involving three or more groups were analyzed by one‐way ANOVA with Tukey's post hoc test. Unpaired Student's t‐tests were used for comparisons between two independent groups. ^*^ *p* < 0.05; ^**^ *p* < 0.01; ^***^ *p* < 0.001; ^****^ *p* < 0.0001; ns, no significance.

To evaluate the therapeutic efficacy of NPs@PGRN in vivo, we administered the nanoparticles to a murine model of chronic knee joint infection. Mice received an intra‐articular injection of a low‐dose bacterial inoculum on day 0, followed by NPs@PGRN treatment starting at day 14. After 2 weeks of therapy, mice were euthanized for endpoint analyses (Figure 6A).

Histopathological analysis (H&E staining) revealed pronounced inflammatory cell infiltration and granuloma formation in both groups. However, NPs@PGRN‐treated mice exhibited significantly greater neutrophil recruitment in periarticular tissues compared to PBS controls, corroborated by elevated peripheral neutrophil counts in blood‐ a clinical hallmark of acute infection resolution (Figure 6B,J). Giemsa staining demonstrated fewer bacteria‐positive regions in NPs@PGRN‐treated tissues (Figure 6B). Collectively, NPs@PGRN therapy markedly attenuated infection severity, evidenced by reduced bacterial burdens, restored body weight, alleviated joint swelling, and improved anemia parameters (Figure 6B–D,J). CFU assays of dissociated periarticular soft tissues confirmed effective control of intracellular bacterial persistence in treated mice (Figure 6E).

The immunosuppressive microenvironment in chronic infections fosters bacterial survival. NPs@PGRN disrupted this pathogenic niche by restoring macrophage immune competence. IHC showed significantly higher IL‐1β levels and reduced PD‐L1 expression in NPs@PGRN‐treated tissues at Day 28 (Figure 6F,G). In vivo flow cytometry revealed enhanced MHC‐II expression and suppressed PD‐L1 on tissue‐resident macrophages, indicating a shift from M2 to M1 polarization (Figure 6H,I). As summarized in Figure 5K, these results underscore NPs@PGRN's dual capacity to rejuvenate macrophage effector functions and reprogram systemic immunosuppressive milieu.

## 3 Discussion

During bacterial infections, as the infection period increases, the local immune microenvironment becomes suppressed, and the function of macrophages is impaired [60, 61, 62, 63]. Modulating the immune functional state of macrophages and reactivating the host immune response are key determinants of the success of infection treatment. In recent years, targeted therapeutic strategies aimed at functionally impaired macrophages and lysosomal repair approaches have emerged as important directions in immunotherapy and infectious disease research [64, 65]. These strategies aim to restore the bactericidal capacity, antigen presentation ability, and inflammatory regulatory functions of macrophages, thereby facilitating pathogen clearance, promoting tissue repair, and controlling chronic inflammation. Approaches include targeting macrophage surface molecules using specific ligands or antibodies, engaging macrophage surface receptors to promote macrophage activation for drug delivery or functional modulation, or reprogramming macrophage polarization states through inflammatory cytokines and modulating macrophage metabolic pathways to regulate their bactericidal functions [22, 66, 67, 68]. In this study, through an extended in vitro intracellular bacterial infection model, we identified synchronized changes in macrophage immune status and lipid metabolism, established their association, and screened out BMP‐a key lipid molecule involved in these changes. In previous studies, BMP has been identified as a lipid predominantly localized to the lysosomal membrane, where it plays a role in maintaining lysosomal functional stability by regulating lipid sorting processes within the lysosome [52, 69]. Further investigation into the literature on BMP revealed that PGRN can bind to BMP and exert regulatory effects [29, 53]. Building upon these foundational studies, we ultimately focused our attention on the direction of lysosomal regulation of macrophage immune function. Through step‐by‐step analysis and integration with the findings of others, we formulated the hypothesis that changes in macrophage immune function are driven by the BMP‐PGRN‐lysosome axis. We then employed a nanomaterial‐based approach to repair lysosomal function and reactivate the inflammatory response capacity of macrophages.

Although the final in vitro and in vivo experimental results demonstrate that this strategy is feasible, several noteworthy issues remain, such as the non‐lysosomal regulatory functions of PGRN. Previous studies have indicated that PGRN, as a multifunctional secretory glycoprotein, is broadly involved in inflammation regulation, tissue repair, neuroprotection, metabolic modulation, and the maintenance of immune homeostasis [70, 71]. PGRN is a key modulator of lysosomal function and plays an important role in the progression of aseptic arthritis through direct anti‐inflammatory actions‐particularly against TNF‐α‐and potential lysosome‐protective effects [72, 73, 74, 75]. Prolonged high levels of PGRN may promote catabolic or immunosuppressive effects through complex signaling networks, necessitating analysis within specific pathological contexts. Based on the lysosomal regulatory functions of PGRN, this study utilizes nanotechnology to deliver PGRN, aiming to repair lysosomal function and reactivate macrophages under infection conditions, thereby offering a novel therapeutic strategy for related diseases.

Moreover, as a growth factor‐like protein, PGRN has been reported to be highly expressed in various malignant tumors. These effects include promoting tumor cell proliferation, inhibiting apoptosis, enhancing invasion and metastasis, polarizing tumor‐associated macrophages (TAMs) toward a pro‐tumor phenotype, and suppressing anti‐tumor immune responses [76, 77]. Therefore, in this study, we adopted a strategy of encapsulating PGRN into nano‐liposomes and delivering it locally into the infected knee joint, aiming to reduce the potential risks of tumorigenesis or neurological disorders associated with systemic delivery of high concentrations of PGRN (e.g., via intravenous injection). Of course, this approach still requires further evaluation in long‐term studies.

As mentioned above, PGRN is a secreted protein with broad anti‐inflammatory effects. To elucidate that the nanoliposomes mediate their effects via the PGRN‐BMP‐lysosome axis, it is necessary to exclude the confounding effects of extracellular PGRN. Under physiological conditions, PGRN binds to the transmembrane receptor Sortilin (SORT1) on the macrophage surface and is internalized via endocytosis to exert its intracellular functions. To rule out potential confounding effects of extracellular PGRN, we pretreated macrophages with a SORT1‐PGRN inhibitor to block the receptor before delivering the PGRN nanoliposomes. Flow cytometry analysis of macrophage polarization states after treatment with the PGRN nanoliposomes further showed that the SORT1 PGRN inhibitor did not noticeably affect the action of NPs@PGRN compared with the untreated group. Taken together, these results support the conclusion that NPs@PGRN restore lysosomal function primarily by delivering the PGRN protein directly to the lysosomal membrane (Figure S7G–I). Bacterial survival plating assays indicated that blocking extracellular PGRN entry did not significantly diminish the therapeutic effect of NPs@PGRN (Figure S7J). Furthermore, a time‐course analysis (0–120 h) of secreted PGRN levels in the supernatant was performed after NPs@PGRN treatment. The results showed no significant changes in secreted PGRN levels compared to the untreated group over 5 days (Figure S7K). In parallel, supernatants collected from the NPs@PGRN‐treated group (0–5 days) were mixed 1:1 with fresh medium and used to culture untreated cells; these conditioned supernatants did not enhance the antibacterial capacity of the normal cells (Figure S7L).

Besides, under prolonged stimulation by intracellular bacteria, macrophages may become excessively inflammatory, leading to a state of “inflammatory exhaustion”. To ensure their own survival, these cells may then shift toward an anti‐inflammatory phenotype. In this context, supplementation with PGRN might partially alleviate the cellular stress associated with survival demands and help restore some of the macrophage's pro‐inflammatory capacity. We view this as a completely novel hypothesis and a distinct research direction that is worthy of further in‐depth investigation. On the other hand, our lipid metabolism results showed that long‐term intracellular bacterial infection led to an increase in BMP lipid content, but the underlying cause of this elevation remains to be further investigated.

In summary, although our preliminary results indicate that the strategy of targeting the PGRN‐BMP‐lysosome axis to regulate macrophage immune function is effective, further exploration is needed to elucidate how long‐term bacterial infection alters BMP levels and to better understand the mechanism of action of this nanosystem.

## 4 Conclusions

This study explored the mechanisms underlying macrophage dysfunction in chronic septic arthritis: prolonged intracellular bacterial infection downregulates PGRN expression, disrupting BMP‐mediated lysosomal homeostasis and fostering an immunosuppressive microenvironment. Key findings include: 1) Temporal lipid metabolic dysregulation in macrophages correlated with progressive immune suppression, with dynamic PGRN‐BMP interaction serving as the central hub governing lysosomal functionality; 2) PGRN‐targeted nanoparticle NPs@PGRN effectively restored lysosomal integrity, reversed M2 polarization, and resuscitated bactericidal capacity; 3) In vivo validation demonstrated NPs@PGRN reduced bacterial burdens and ameliorated tissue pathology through neutrophil infiltration activation and pro‐inflammatory cytokine secretion. The developed NPs@PGRN nanoparticles offered a combined approach with both antimicrobial effects and immune microenvironment modulation, showing potential for the treatment of chronic septic arthritis.

## 5 Experimental Section

Detailed experimental procedures are provided in the Supplementary Materials.

### 5.1 Statistical Analysis

Statistical analyses were performed using GraphPad Prism 9. Data are presented as mean ±SD. Comparisons involving three or more groups were analyzed by one‐way ANOVA with Tukey's post hoc test. Unpaired Student's t‐tests were used for comparisons between two independent groups. All p‐values were two‐tailed, with statistical significance defined as *p* < 0.05. Significance levels are denoted as follows: ^*^ *p* < 0.05, ^**^ *p* < 0.01, ^***^ *p* < 0.001, ^****^ *p* < 0.0001. ns indicates non‐significance.

## Conflicts of Interest

The authors declare no conflicts of interest.

## Supporting information




**Supporting File**: advs74713‐sup‐0001‐SuppMat.docx.

## Data Availability

The data that support the findings of this study are available from the corresponding author upon reasonable request.
