# Supplementary material for: Targeting the PGRN‐BMP Lysosomal Axis With NPs@PGRN Reverses Immunometabolic Dysfunction in Chronic Septic Arthritis
Source: Adv Sci (Weinh). 2026 Mar 16;13(28):e12133. doi: 10.1002/advs.202512133 (PMC13185817; doi:10.1002/advs.202512133)
Supplement: Supplementary file 1 — Supporting File: advs74713‐sup‐0001‐SuppMat.docx. [file ADVS-13-e12133-s001.docx]

**Supporting Information**

**S1. Supplementary figures**

**S2. Experimental section**

**S2.1** **Materials and reagents**

**S2.2 Materials synthesis and characterization**

**S2.3 Biological experimental designs *in vitro* and *in vivo***

**S1. Supplementary figures**

**
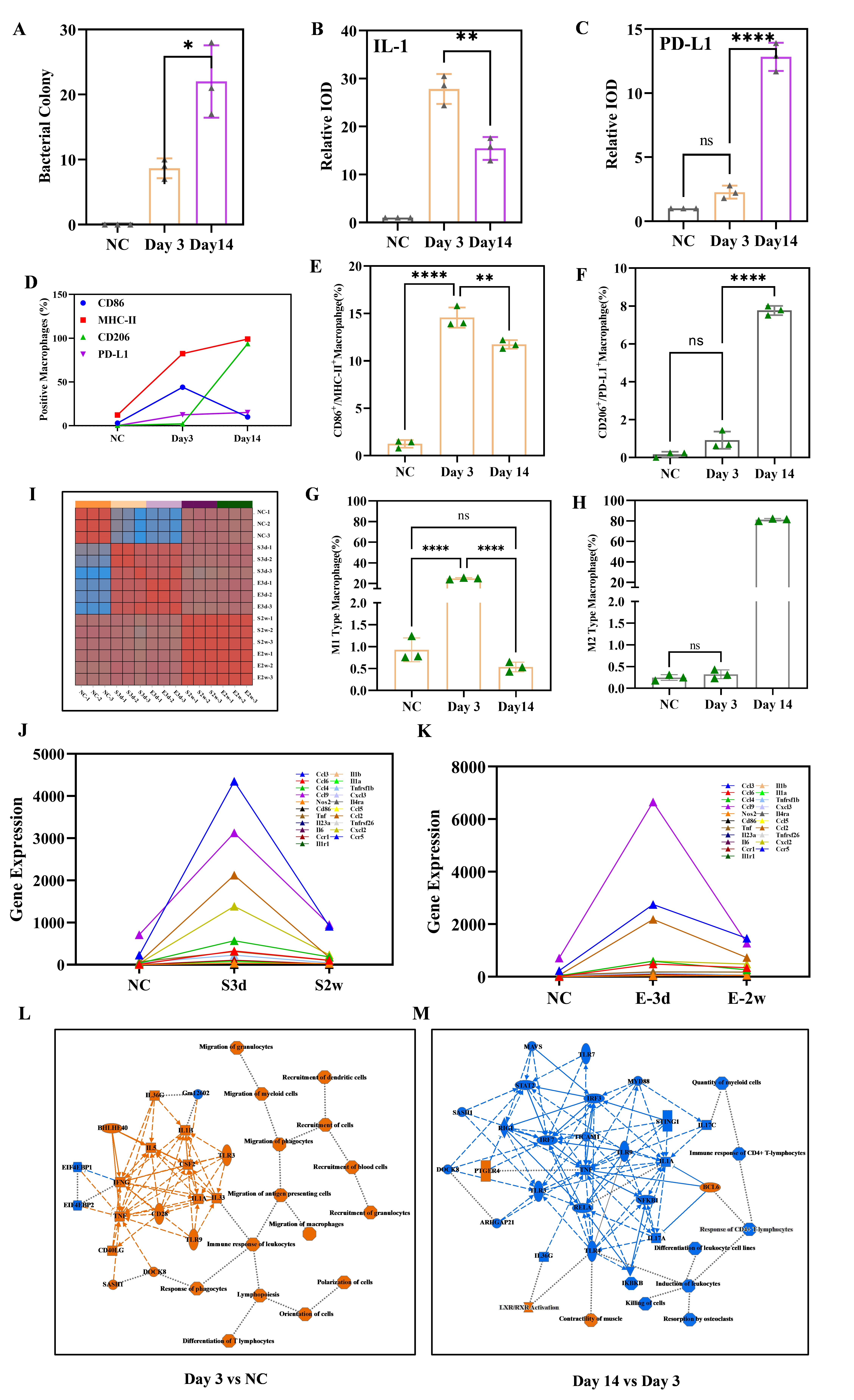
**

**Supplementary Figure S1** A. Bacterial colony counting statistics from Giemsa staining in Figure 1C (mean ± SD n = 3). B, C. Quantitative analysis of IL-1 and PD-L1 expression from IHC staining in Figure 1D (mean ± SD n = 3). D. Quantitative analysis of flow cytometry results from Figure 1E (mean ± SD n = 3). E-H. Quantitative analysis of flow cytometry data from Figure 1F (mean ± SD n = 3). I. Correlation heatmap of samples from different infection conditions. J, K. Expression trend statistics of inflammatory genes from Figure 1J. L, M. Graphical summary of *E. coli-*infected group IPA analysis. Statistical significance was determined by one-way ANOVA followed by Tukey's post hoc test. **P* < 0.05; ***P* < 0.01; ****P* < 0.001; *****P* < 0.0001; ns, no significance.


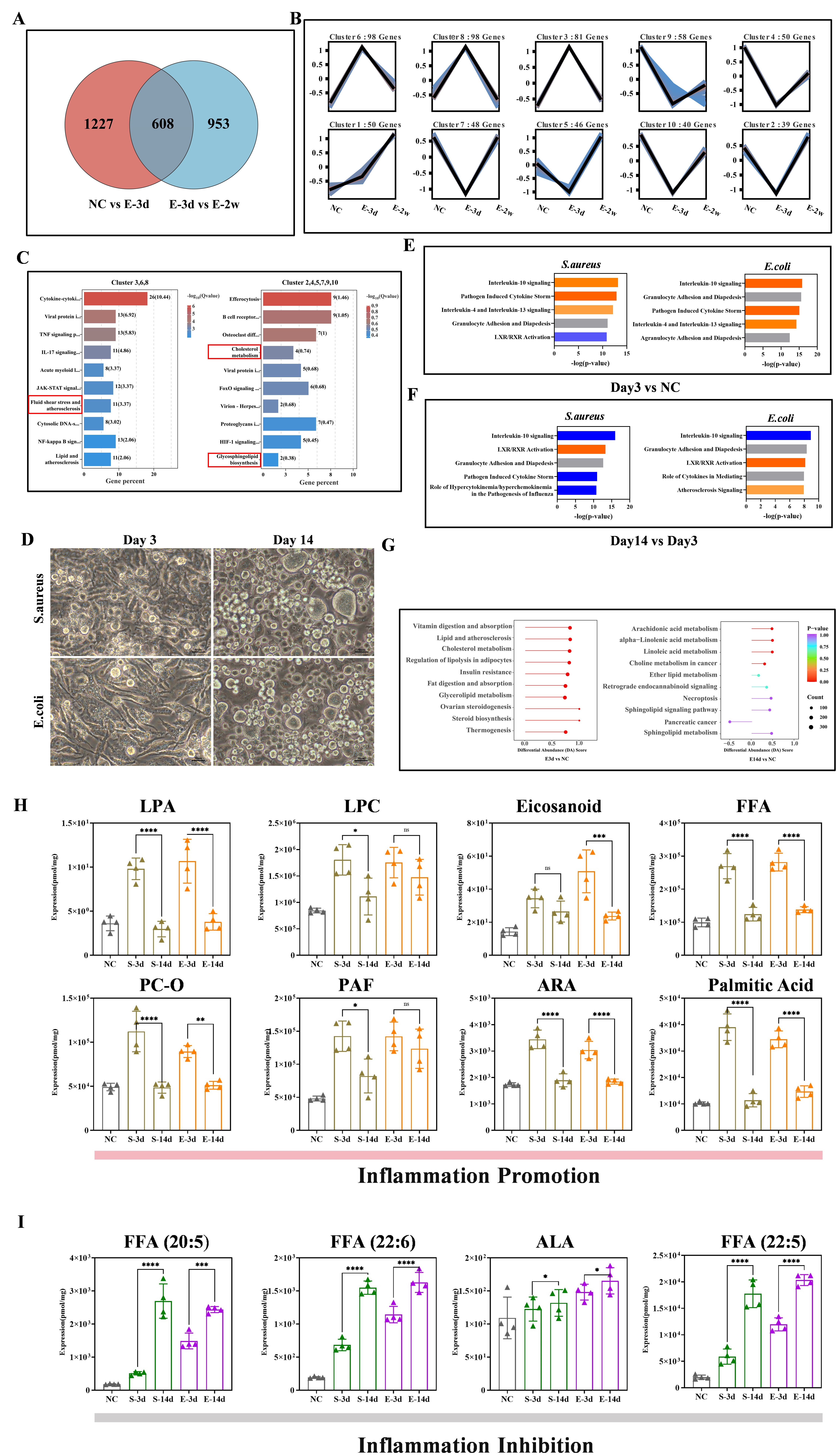


**Supplementary Figure S2** A. Venn diagram of DEGs in E. coli-infected groups. B. Trend analysis of 608 DEGs from Figure S1A. C. GO enrichment of merged clusters from Figure S1B. D. Morphological changes in RAW264.7 cells during intracellular bacterial infection. E, F. IPA pathway analysis of *S. aureus*-and *E. coli-* infected group. G. Pathway enrichment of lipidomics in *E. coli*-infected group. H, I. Temporal trends of pro- and anti-inflammatory lipids in macrophages during persistent infection (mean ± SD n = 4 per group). Statistical significance was determined by one-way ANOVA followed by Tukey's post hoc test. **P* < 0.05; ***P* < 0.01; ****P* < 0.001; *****P* < 0.0001; ns, no significance.


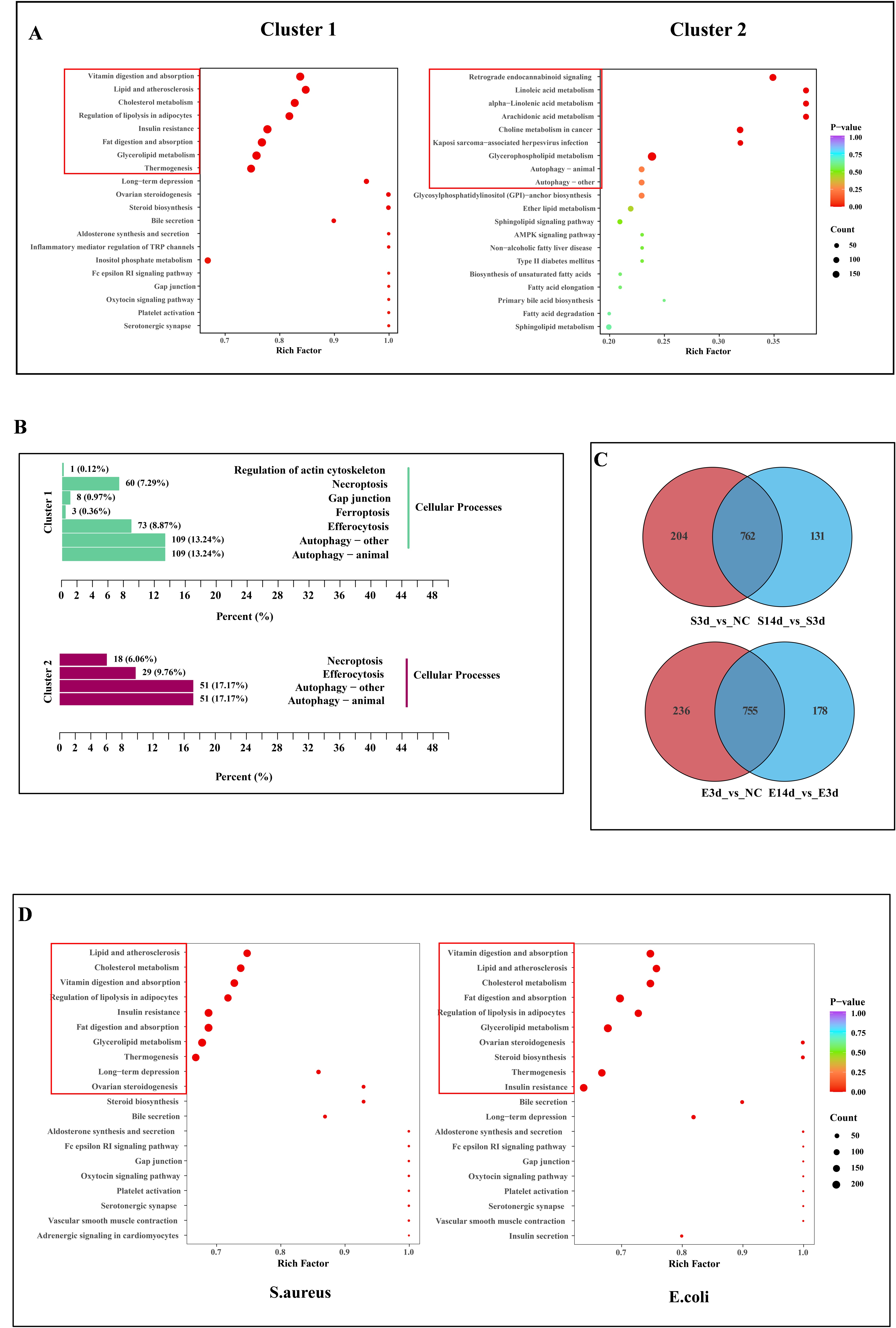


**Supplementary Figure S3** A. Pathway enrichment analysis of lipids from K-means clustering in Figure 2K. B. Cellular function enrichment of K-means clustered lipids from Figure 2K. C. Venn diagram of differentially regulated lipids. D. Top 20 enriched pathways for 762 and 756 differential lipids identified in Figure S3C.

**
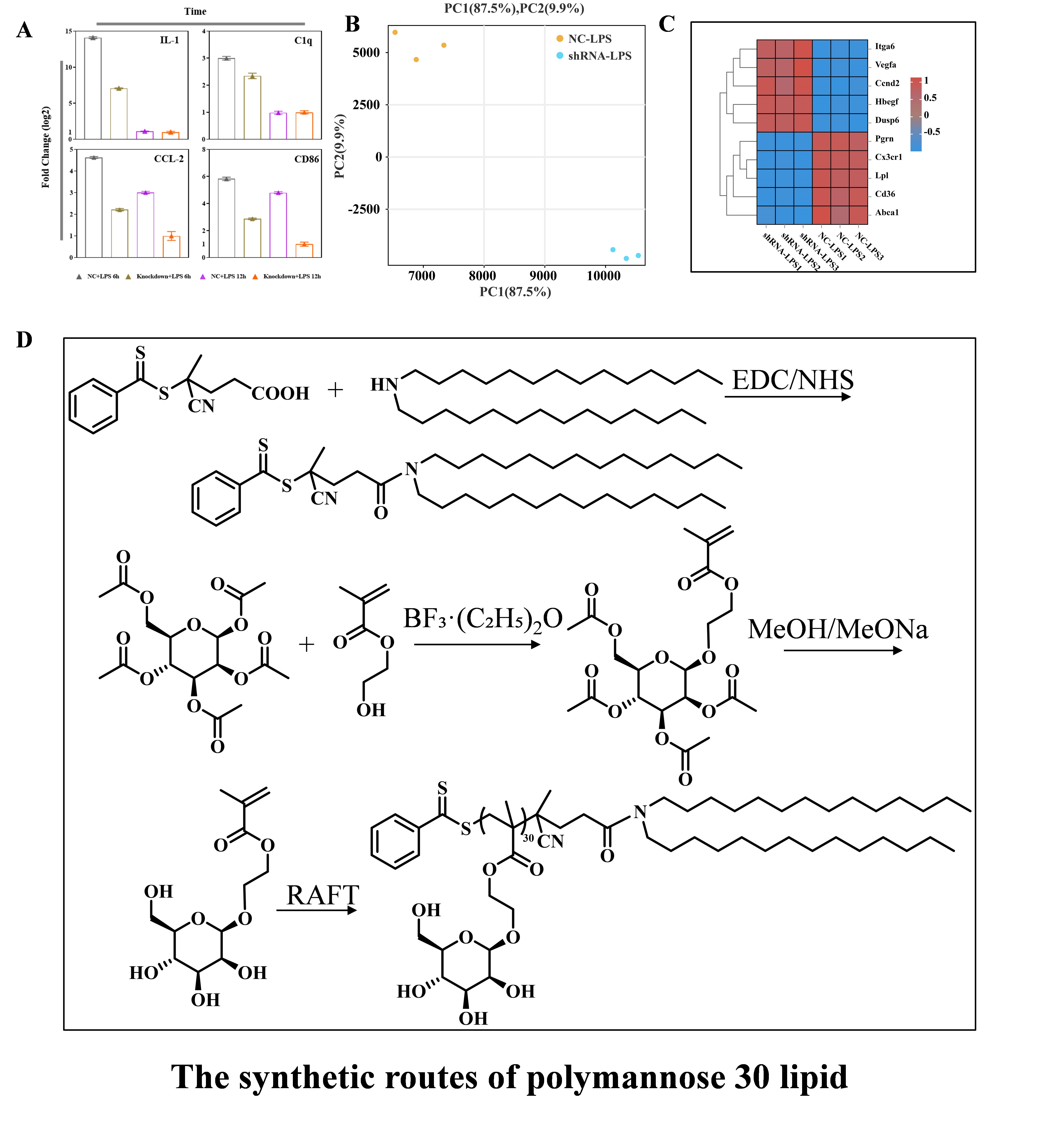
**

**Supplementary Figure S4** A. qPCR analysis of inflammation-related genes in macrophages under LPS stimulation (PGRN-knockdown vs NC, both of two groups were treated with LPS). B. PCA analysis of RNA-seq data from LPS-stimulated wild-type vs PGRN-knockdown macrophages. C. Heatmap of top 10 DEGs from RNA-seq analysis of LPS-stimulated wild-type vs PGRN-knockdown macrophages. D. Synthesis scheme of macrophage-targeting PM30 nanoparticles (mean ± SD n = 3). Statistical significance was determined by one-way ANOVA followed by Tukey's post hoc test. **P* < 0.05; ***P* < 0.01; ****P* < 0.001; *****P* < 0.0001; ns, no significance.


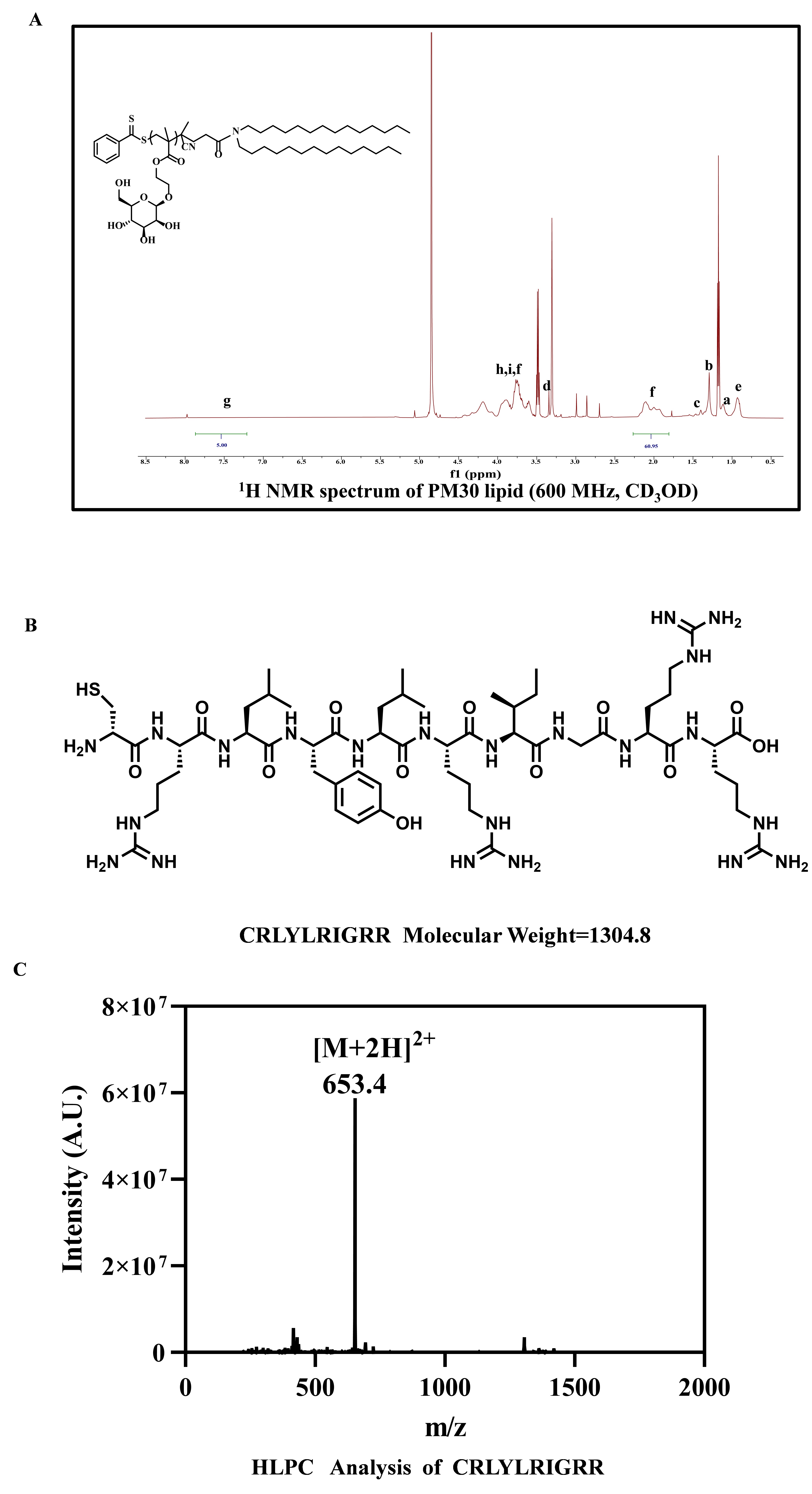


**Supplementary Figure S5** A. Proton nuclear magnetic resonance (^1^H NMR) spectrum of PM30. B. Molecular structure of the bacteria-targeting peptide CRLYLRIGRR. C. HLPC analysis of CRLYLRIGRR.


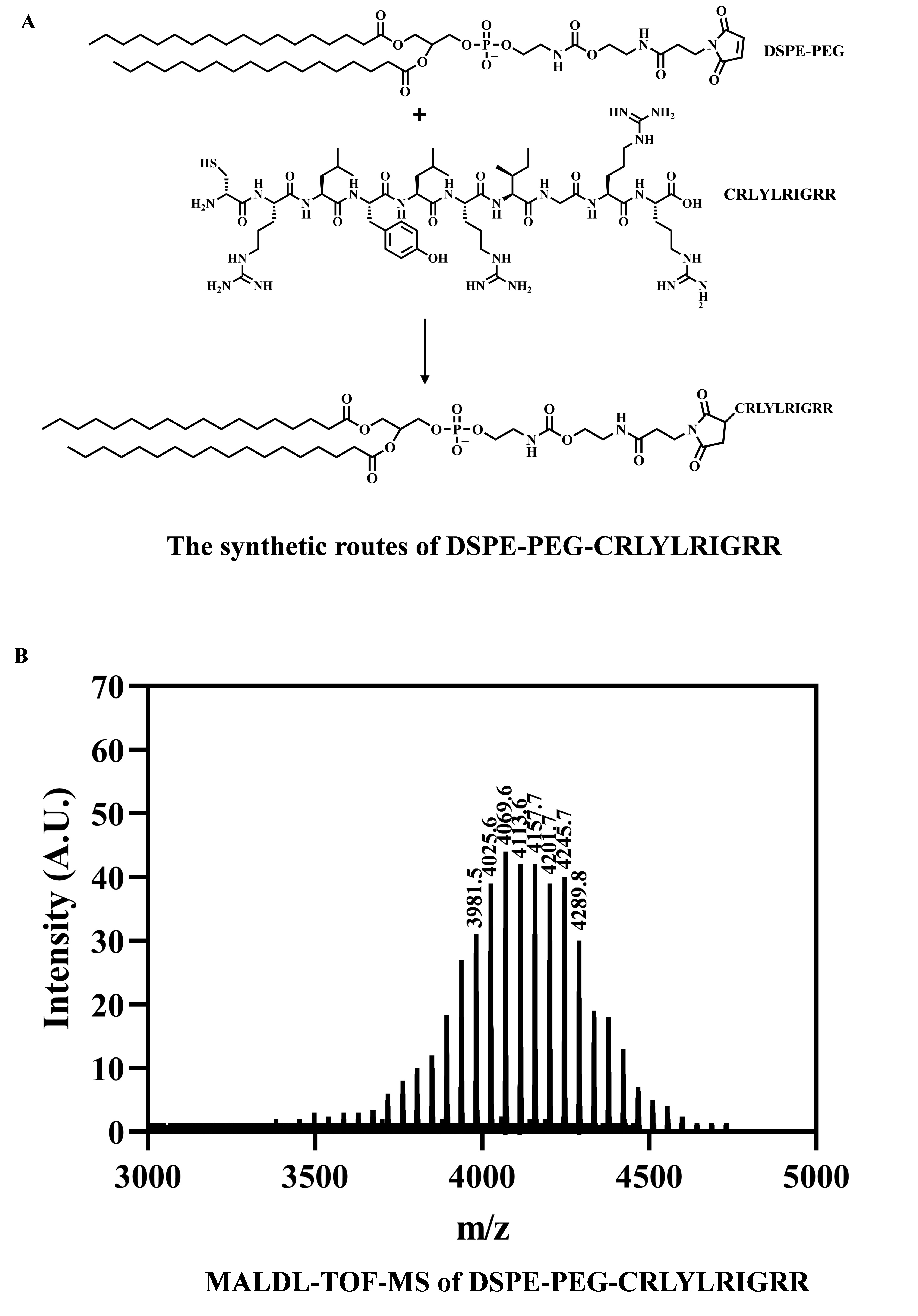


**Supplementary Figure S6** A. Schematic of the DSPE-PEG-CRLYLRIGRR synthesis route. B. MALDI-TOF-MS analysis of DSPE-PEG-CRLYLRIGRR.

**
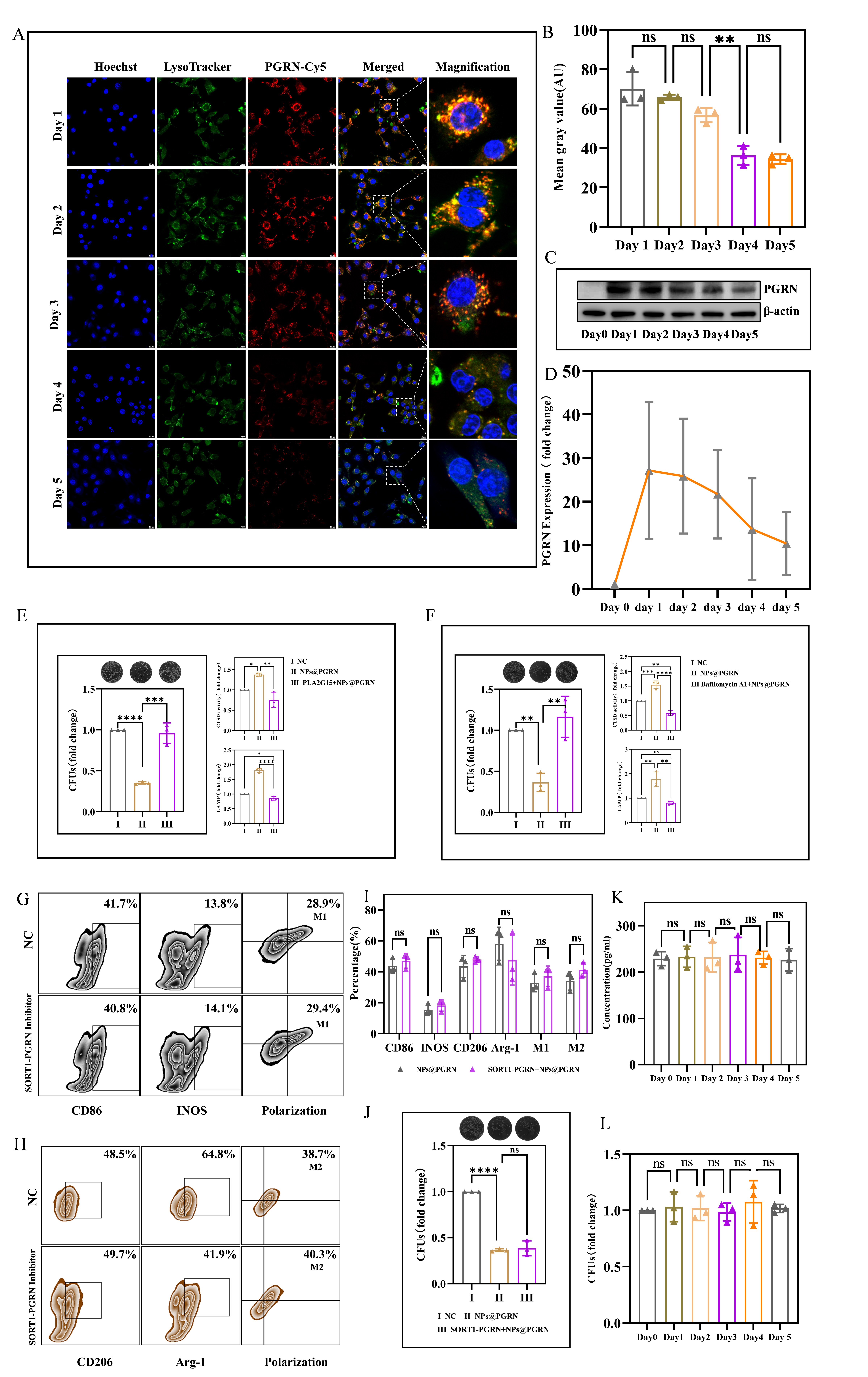
**

**Supplementary** **Figure S7** A. Time-course analysis of PGRN colocalization with lysosomes (0-5 days) following NPs@PGRN-Cy5 delivery in PGRN-knockdown macrophages. B. Quantitative analysis of PGRN‑Cy5 fluorescence intensity (mean ± SD n = 3). C.  Intracellular PGRN levels within 5 days after nanoliposome‑mediated delivery in PGRN-knockdown macrophages. D. Quantification of figure C (mean ± SD n = 3). E. Bacterial killing capacity, lysosomal function, and lysosomal membrane permeability after NPs@PGRN delivery in PGRN-knockdown macrophages pretreated for 12 h with the BMP hydrolase PLA2G15 (mean ± SD n = 3). F. Bacterial killing capacity, lysosomal function, and lysosomal membrane permeability after NPs@PGRN delivery in PGRN-knockdown macrophages pretreated for 12 h with bafilomycin A1(mean ± SD n = 3). G-I. Changes in macrophage polarization after NPs@PGRN delivery in PGRN-knockdown cells pretreated with the SORT1‑PGRN inhibitor (mean ± SD n = 3). J. Antibacterial effect of NPs@PGRN treatment in PGRN-knockdown cells pretreated with the SORT1‑PGRN inhibitor (mean ± SD n = 3). K. Extracellular PGRN levels in the supernatant over 5 days following NPs@PGRN treatment in PGRN-knockdown cells (mean ± SD n = 3). L. Effect of supernatant collected over 5 days respcetively after NPs@PGRN treatment on the antibacterial function of untreated PGRN-knockdown macrophages (mean ± SD n = 3). Statistical significance was determined by one-way ANOVA followed by Tukey's post hoc test. **P* < 0.05; ***P* < 0.01; ****P* < 0.001; *****P* < 0.0001; ns, no significance.

**S2. Experimental section**

**S2.1 Materials and reagents**

High-glucose DMEM medium, fetal bovine serum (FBS), phosphate-buffered saline (PBS), and Triton X-100 lysis buffer were purchased from Thermo Fisher Scientific (Shanghai, China). Antibodies for flow cytometry analysis, Cyto-Fast™ Fixation/Permeabilization Buffer Kit, and True-Nuclear™ Transcription Factor Buffer Kit were obtained from BioLegend (San Diego, CA, USA), unless otherwise specified. Antibodies for Western blot (WB) analysis, including anti-TNF-α, anti-IL-1β, anti-NLRP3, anti-LAMP-2, anti-Galectin-3, anti-β-actin, and corresponding secondary antibodies, were procured from Proteintech Biotechnology (Wuhan, China). 4',6-Diamidino-2-Phenylindole (DAPI) was acquired from Beyotime Biotechnology (Shanghai, China). PGRN-specific plasmids for constructing stable macrophage cell lines were provided by Vigenebio Biotechnology Co., Ltd. (Shandong, China). Lipid BMP was purchased from Sigma-Aldrich (St. Louis, MO, USA), and recombinant progranulin (PGRN) protein was obtained from Proteintech Biotechnology (Wuhan, China). Recombinant M-CSF was purchased from Hangzhou Yangming Biotechnology Co., Ltd (Hangzhou, China). Lysotracker™ Red for lysosomal labeling was acquired from Beyotime Biotechnology (Shanghai, China), while fluorescent reagents for lysosomal functional assays (DQ-BSA assay) were procured from Thermo Fisher Scientific (Shanghai, China). RAW 264.7 macrophages were obtained from the Cell Collection of Chinese Academy of Sciences (Shanghai, China). *S.aureus* and *E.coli* were purchased from the American Type Culture Collection (ATCC). Male BALB/c mice (10 weeks old) were purchased from the Vital River Laboratory Animal Technology Co. (Beijing, China), and the animal experiments were approved by the Animal Ethics Committee of the Second Affiliated Hospital of Zhejiang University School of Medicine.

**S2.2 Materials synthesis and characterization**

**Synthesis of Polymannose 30 Lipids**

Synthesis of N, N-Ditetradecyl Chain Transfer Agent: In a 100 mL single-neck flask, N, N-ditetradecylamine (800 mg, 1.96 mmol) and the chain transfer agent 4-cyano-4-(thiobenzoylthio) pentanoic acid (313 mg, 1.12 mmol) were dissolved in anhydrous dichloromethane. EDC (248.3 mg, 1.3 mmol) and DMAP (20 mg, 0.16 mmol) were then added, and the mixture was stirred at room temperature overnight. Post-reaction, the solution was washed three times with saturated brine, dried over anhydrous sodium sulfate, filtered, and concentrated under reduced pressure to yield a dark red oily crude product. Purification via column chromatography (dichloromethane/methanol = 20/1, v/v) followed by solvent removal under reduced pressure afforded the N, N-ditetradecyl chain transfer agent (455.6 mg, 68% yield) as a dark red oily liquid.

Synthesis of HEMA-Mannose Monomer: Pentaacetyl mannose (5 g, 12.8 mmol) and 2-hydroxyethyl methacrylate (HEMA, 1.95 g, 15 mmol) were dissolved in anhydrous dichloromethane under nitrogen atmosphere and cooled in an ice bath. Boron trifluoride diethyl etherate (10 mL, 78.8 mmol) was added dropwise. After removing the ice bath, the reaction proceeded at room temperature overnight under nitrogen. The mixture was washed with saturated sodium bicarbonate, dried over anhydrous sodium sulfate, and purified via column chromatography (dichloromethane/ethyl acetate = 20/1, v/v) to yield acetylated HEMA-mannose (5.06 g, 85.9% yield) as a colorless oil. Deprotection was achieved by dissolving acetylated HEMA-mannose (3 g, 6.5 mmol) in anhydrous methanol containing 0.03 M sodium methoxide, stirring for 30 minutes. Reaction progress was monitored by TLC (acetonitrile/water = 9/1, v/v). After neutralization with DOWEX® 50WX8 ion-exchange resin, filtration, and solvent removal, deprotected HEMA-mannose (1.46 g, 76.9% yield) was obtained as a colorless oil.

Synthesis of Polymannose Lipids: In a 10 mL flask, HEMA-mannose monomer (228 mg, 0.78 mmol), AIBN (20 mg, 0.12 mmol), and N, N-ditetradecyl chain transfer agent (17.4 mg, 0.026 mmol) were dissolved in DMF, degassed under nitrogen for 30 minutes, and heated at 65°C overnight. The reaction was quenched in liquid nitrogen, precipitated in ice-cold diethyl ether, dissolved in anhydrous methanol, and reprecipitated three times to yield polymannose lipids as a white solid (270 mg). The product was vacuum-dried for 24 hours, characterized by ¹H-NMR.

**Synthesis of Bacterial-Targeting Peptide**

The Bacterial-Targeting Peptide, CRLYLRIGRR, was synthesized via solid-phase synthesis. Successful synthesis was confirmed by mass spectrometry (MS) and high-performance liquid chromatography (HPLC). DSPE-PEG-maleimide (DSPE-PEG-MAL, 100 mg, 0.034 mmol) and CRLYLRIGRR peptide (49.0 mg, 0.037 mmol) were dissolved in DMSO, followed by the addition of triethylamine (TEA, 3.8 mg, 0.034 mmol). The reaction proceeded under nitrogen atmosphere at room temperature for 24 hours. The product was dialyzed against deionized water using dialysis tubing with a molecular weight cutoff (MWCO) of 1000 Da for 48 hours, lyophilized to yield a white powder (107.5 mg), and validated by MALDI-TOF mass spectrometry to confirm successful synthesis of DSPE-PEG-CRLYLRIGRR.

**Synthesis of PGRN-Cy5**

First, 20 μg of PRGN protein was dissolved in 200 μL of PBS (pH 7.4) buffer. The Cy5-NHS fluorescent dye was then dissolved in DMSO to prevent quenching of the active groups by water, with its concentration adjusted to 10 mg/mL. Subsequently, the dye solution was slowly added to the protein solution at a molar ratio of 1:5 (fluorescent dye to protein). The reaction mixture was stirred overnight at room temperature under light-protected conditions. After completion of the reaction, unbound free dye was removed by dialysis over 24 hours (with the dialysis solution replaced three times during this period). Finally, the Cy5-labeled PRGN protein was obtained by freeze-drying.

**Nanoparticle Preparation**

A lipid mixture containing phosphatidylethanolamine (PE, 10 mg), cholesterol (2 mg), DSPE-PEG-CRLYLRIGRR (0.2 mg), and PM30 (0.2 mg) was dissolved in 2 mL chloroform. A thin lipid film was formed via rotary evaporation under reduced pressure. The film was vacuum-dried for 12 hours, rehydrated with 10 mL PBS containing 1 mg protein, and sonicated (probe sonicator, 10 minutes) to form protein-encapsulated liposomes.

**Encapsulation Efficiency Determination**

Encapsulation efficiency was indirectly quantified via ultracentrifugation. A 1 mL sample was centrifuged at 16,000 rpm (4°C, 20 minutes) to pellet free protein. The supernatant was collected, and free protein concentration was measured using the Bradford assay. The loading rate of PGRN protein is 20-30%.

**TEM Observation of Nanoparticle-Bacterial Interactions**

Prepared nanoparticles were resuspended in double-distilled water (ddH₂O) and co-incubated with or without bacteria for 30 minutes. A small aliquot was applied to a copper grid, allowing full contact between the nanoparticle suspension and the grid. Excess suspension was removed, followed by negative staining with uranyl acetate (1% w/v) for 1-2 minutes. Residual stain was blotted away, and the grid was air-dried prior to TEM imaging.

**Nanoparticle Phagocytosis Assay**

Fluorescently labeled NPs@PGRN were prepared for phagocytosis evaluation. RAW264.7 macrophages were seeded into 6-well plates and incubated with Fluorescently labeled NPs@PGRN (2.5mg/mL) in culture medium. After co-incubation for 30 minutes, cells were washed three times with PBS, fixed, and counterstained with DAPI (5 minutes). Cellular uptake was visualized using fluorescence microscopy.

**S2.3 Biological experimental designs *in vitro* and *in vivo***

**Cell Culture and Intracellular Bacterial Infection Model Establishment**

RAW264.7 macrophages were cultured in high-glucose DMEM supplemented with 10% fetal bovine serum at 37°C under 5% CO₂. The medium was replaced every 48 hours, and cells were subcultured at 80% confluency.

Isolation and Culture of bone marrow-derived macrophages: Healthy 6-8-week-old BALB/c mice were euthanized and then dissected under aseptic conditions in a biological safety cabinet. After removing hair and muscle tissue, the mouse tibia were extracted and placed in a culture dish containing an appropriate amount of PBS. The harvested tissue was minced, and the marrow cavity was flushed thoroughly using a 1 mL syringe filled with PBS containing dual antibiotics (penicillin-streptomycin). The bone marrow suspension was then centrifuged at 1000 rpm for 5 minutes. The supernatant was discarded, and the cell pellet was resuspended in red blood cell lysis buffer for 10 minutes. After processing, the cells were centrifuged again at 1000 rpm for 5 minutes and resuspended. The resulting cells were subsequently seeded into pre-prepared culture media: for bone marrow-derived macrophages, the cells were resuspended in RPMI-1640 medium supplemented with 10% fetal bovine serum, 1% penicillin-streptomycin dual antibiotics, and 40 ng/mL M-CSF. The medium was replaced every 48 hours, and cells were subcultured at 80% confluency.

To establish the *in vitro* intracellular infection model, macrophages were seeded in 6-well plates and infected with Staphylococcus aureus at a multiplicity of infection (MOI) of 50 for 1 hour. Following infection, cells were thoroughly washed with PBS and treated with gentamicin (20 mg/L) for 2 hours to lyse residual extracellular bacteria. After additional PBS washes, cells were maintained in fresh medium containing low-dose gentamicin (5mg/L) to suppress extracellular bacterial growth while preserving intracellular bacterial viability.

**Assessment of Macrophage Phagocytic Capacity and Intracellular Bacterial Clearance**

RAW264.7 macrophages were seeded in 6-well plates and infected with GFP-labeled *S.aureus* to establish the intracellular infection model. After designated treatments, cells were collected and analyzed by flow cytometry to quantify GFP signal intensity, reflecting intracellular S. aureus loads.

Phagocytic capacity was assessed by flow cytometry 1hour post-infection. To evaluate intracellular bacterial clearance, cells were further incubated in medium containing gentamicin (5 mg/L) for 48 hours, followed by flow cytometric detection of residual intracellular bacteria. The intracellular bacterial clearance capacity was evaluated by comparing GFP-positive cell proportions before and after incubation.

**Construction of PGRN shRNA Knockdown Macrophages**

Cell Plating: Select appropriate culture dishes and seed 293T host cells one day prior to experiments, achieving 70-80% confluency. Transfection: Mix the PGRN lentiviral vector (pLKO.1-U6-PURO) with packaging plasmids (psPAX2, pMD2.G) and transfection reagent (Lipofectamine 2000) according to the manufacturer’s recommended ratios. Add the transfection mixture to the culture dish and gently mix to ensure uniform distribution. Incubate for 4-6 hours, then replace the transfection medium with fresh complete medium. Viral Harvest: Collect virus-containing supernatant 48-72 hours post-transfection. Filter the supernatant through a 0.45 μm membrane to remove cellular debris. Concentrate the viral particles via ultracentrifugation and store in low-retention cryovials at -80°C.Cell Infection: Seed RAW264.7 cells in logarithmic growth phase into 12-well plates at 1×10⁵ cells/mL and incubate overnight. Replace medium with fresh medium containing PBS-diluted viral supernatant. Refresh medium and viral supernatant every 24 hours for 72 hours. Selection and Validation: Select stable knockdown cells via puromycin (PURO) resistance screening. Validate knockdown efficiency by qPCR (≥80% efficiency required) and freeze qualified cell lines for future use. For primary mouse BMDMs, AAV virus was constructed to knockdown PGRN in cells. The shRNA targeting sequence of PGRN: ACTCATCCTGAGTCACCCTAT

**RNA-seq and Targeted Lipidomics**

In RAW264.7 cells, reverse time-course intracellular bacterial infection models were established at 3 and 14 days, with the NC group remaining uninfected. For RNA-seq, macrophages were collected at Day 14 using cell scrapers. Total RNA was extracted using TRIzol reagent and immediately flash-frozen in liquid nitrogen. Samples were submitted to OE Biotech Co., Ltd. (Shanghai, China) for RNA sequencing. For targeted lipid metabolomics, cells were rapidly frozen in liquid nitrogen at Day 14 and sent to MetWare Biotechnology Co., Ltd. (Wuhan, China) for analysis.

**Observation of lysosome by TEM**

To observe lysosomal structures in macrophages via transmission electron microscopy (TEM), adherent cells were collected and centrifuged into pellets, followed by dual fixation with 2.5% glutaraldehyde (4°C, 24 hours) and 1% osmium tetroxide (4°C, 1–2 hours; note: osmium tetroxide handling required ventilation) to preserve ultrastructural integrity. Fixed samples were washed with phosphate buffer, dehydrated through an ethanol gradient (50% to 100%), infiltrated with epoxy resin, and embedded for polymerization at 60°C to form hardened blocks. Trimmed resin blocks were ultrathin-sectioned (50–70 nm) using an ultramicrotome, mounted on Formvar-coated copper grids, and sequentially stained with uranyl acetate and lead citrate. Stained grids were imaged under optimized parameters using a transmission electron microscope.

**Lysosome Labeling and** **Lysosome Function Detection**

Lysotracker binds to acidic lysosomes and emits fluorescence. Add the reagent to prepared cells at the concentration specified in the manufacturer’s instructions. Incubate in the dark for 5 minutes, then wash off residual reagent with PBS before observation. DQ-BSA-Green assay was applied for Lysosome Function Detection. Add the fluorescent reagent to cells from different treatment groups at the recommended concentration (as per the protocol). Incubate in the dark for 30 minutes, wash with PBS to remove excess reagent, and observe under a fluorescence microscope. Lysotracker Kit (C1064) was purchased from Beyotime, China. DQ-BSA-Green Assay Kit (PC4009) was purchased from Shanghai Shuji Biotechnology, China.

**Lysosomal β-Galactosidase Activity Assay**

Lysosomal β-Galactosidase Staining Kit (C0605, Beyotime, China) was used for test. For cells cultured in 6-well plates, remove the culture medium and wash the cells once with PBS or HBSS. Add 1 mL of β-galactosidase staining fixative solution, and incubate at room temperature for 15 minutes. Remove the fixation solution, and wash the cells three times with PBS (3 minutes each time). Remove the PBS, and add 1 mL of staining working solution to each well. Incubate at 37°C overnight, then observe under a light microscope.

**Lysosomal Cathepsin D Activity Assay**

Cathepsin D Activity Assay Kit (Fluorometric, ab65302, Abcam, USA) was used to performed the test. According to the protocol, cells accepted NPs treatment were lysed by the solution supplied in the Kit. Add reaction mix to samples and incubate for 1-2 hours at 37°C. Readout on any fluorometric (Ex/Em 328/460 nm) plate reader and analyze. The data were normalized and transformed into log₂ fold values for analysis.

**Lysosomal LMP Assay**

LMP Assay was performed using Acridine Orange Staining Kit (C0233S, Beyotime, China). Gently aspirate the culture medium from the wells. Wash the wells with PBS (C0221A) for approximately 10 seconds, then aspirate the PBS. Add the acridine orange staining solution, incubate at 37°C for 2–10 minutes, then aspirate the staining solution. Wash the wells with PBS for about 10 seconds. Repeat the washing step once.Add an appropriate amount of cell culture medium, staining buffer, or other suitable solution to cover the bottom of the wells. Place the plate under a microscope for observation. Detect fluorescence intensity using a fluorescence microplate reader with its bottom-read function, at excitation/emission wavelengths of approximately Ex/Em = 530/640 nm. The data were normalized and transformed into log₂ fold values for analysis.

**Knee Joint Infection Model Establishment**

Mice were housed under standardized conditions: constant temperature (21 ± 2°C), 12-hour light/dark cycles, ad libitum access to water and irradiated food, and pathogen-free environment with humidity maintained at 45-65%. Ten-week-old male BALB/c mice were purchased from Vital River Laboratory Animal Technology Co., Ltd. (Beijing, China). All procedures complied with animal research reporting guidelines and were approved by the Animal Ethics Committee of the Second Affiliated Hospital, Zhejiang University School of Medicine. For surgery, mice were anesthetized via nasal cone with 1-2% isoflurane in oxygen. A 10 µL bacterial suspension (10⁸ CFU/mL Staphylococcus aureus) was intra-articularly injected using a microsyringe. Euthanasia was performed via CO₂ asphyxiation.

**Evaluation of Local Immune Microenvironment Changes *In Vivo***

To assess immune microenvironment alterations in periarticular tissues and macrophage functional dynamics post-bacterial infection, knee joint soft tissues were harvested at Day 3 and Day14 post-infection. The collected periarticular knee tissues were subjected to H&E staining, Giemsa staining, and immunohistochemical (IHC) staining to evaluate tissue immune responses, inflammatory infiltration, and bacterial distribution. For flow cytometry analysis, tissues were minced with surgical scissors, digested in tissue lysis buffer at 37°C for 2 hours, and filtered through a 70 μm cell strainer. Isolated cells were washed and subjected to flow cytometry for functional profiling. For detailed immunophenotyping, single-cell suspensions were pre-blocked with Fc receptor blocking antibody (BioLegend, San Diego, CA, USA; 1:100 dilution) at room temperature for 10 minutes. Cells were stained with Zombie viability dye and surface antibodies (CD45, CD11b, F4/80, CD86, MHC-II, PD-L1; BioLegend, dilutions per manufacturer’s protocol). For intracellular CD206 staining, cells were fixed/permeabilized using the True-Nuclear™ Transcription Factor Buffer Kit (BioLegend) after surface staining, followed by anti-CD206 antibody incubation (BioLegend, protocol-specified dilution). Flow cytometry was performed on a Beckman Coulter CytoFLEX LX system with CytExpert v2.4 software. Data were analyzed using FlowJo v10 (BD Biosciences, San Jose, CA, USA).

**Evaluation of Nanomaterial Efficacy *In Vivo***

As previously described, a murine knee joint infection model was established, with treatment initiated at Day 14. The experimental group received an intra-articular injection of 50 μL NPs@PGRN (2.5 mg/mL), while controls received an equivalent volume of PBS. Mice were euthanized at Day 21, as mentioned above, the collected periarticular knee tissues were subjected to H&E staining, Giemsa staining, and immunohistochemical (IHC) staining to evaluate tissue immune responses, inflammatory infiltration, and bacterial distribution. For intracellular bacteria counting, periarticular tissues were harvested, minced, digested in tissue lysis buffer at 37°C for 2 hours, and filtered through a 70 μm strainer. Isolated cells were washed and analyzed by flow cytometry to assess macrophage functional alterations. Macrophages were flow-sorted and incubated in medium containing gentamicin (20 mg/L) for 2 hours to eliminate extracellular bacteria. Cells were lysed with Triton X-100, and lysates were serially diluted, plated on Mueller-Hinton agar plates, and incubated overnight at 37°C for colony enumeration.
